# Transketolase (TKT) activity and nuclear localization promote hepatocellular carcinoma in a metabolic and a non-metabolic manner

**DOI:** 10.1186/s13046-019-1131-1

**Published:** 2019-04-11

**Authors:** Zhaoyu Qin, Chan Xiang, Fan Zhong, Yang Liu, Qiongzhu Dong, Kai Li, Wenhao Shi, Chen Ding, Lunxiu Qin, Fuchu He

**Affiliations:** 1 Institutes of Biomedical Sciences, Fudan University, Shanghai, 200032 China; 2 Department of Pathology, Shanghai Chest Hospital, Shanghai Jiao Tong University, Shanghai, 200030 China; 3 Department of Surgery, Huashan Hospital, Cancer Metastasis Institute, Fudan University, Shanghai, 200040 China; 4 State Key Laboratory of Proteomics, Beijing Proteome Research Center, National Center for Protein Sciences, Beijing, 102206 China

**Keywords:** Hepatocellular carcinoma, Pentose phosphate pathway, Transketolase, Nuclear localization, Non-metabolic function

## Abstract

**Background:**

Metabolic reprogramming is one of the hallmarks of cancer cells. The pentose phosphate pathway (PPP), a branch of glycolysis, is an important metabolic pathway for the survival and biosynthesis of cancer cells. Transketolase (TKT) is a key enzyme in the non-oxidative phase of PPP. The mechanistic details of TKT in hepatocellular carcinoma (HCC) development remain unclear.

**Methods:**

TKT level and subcellular location were examined in HCC cell lines and tissue samples. We established the TKT overexpression and knocking-down stable cells in HCC cell lines. Proliferation, migration, viability and enzyme activity assays in vitro, tumor growth and metastasis assays in vivo were employed to test the effects of TKT on HCC development. GFP-tagged TKT truncations and mutants were used to locate the nuclear localization sequence (NLSs) of TKT. Cross-linking co-IP/MS was applied to identify the interaction proteins of nuclear TKT.

**Results:**

We showed that TKT increased the proliferation and migration of HCC cells, as well as the viability under oxidative stress in vitro and accelerated the growth and metastasis of HCC cells in vivo. We found as a key enzyme of PPP, TKT could promote the proliferation, cell cycle, migration and viability by regulating the metabolic flux. Moreover, it was firstly reported that unlike other key enzymes in PPP, TKT showed a strong nuclear localization in HCC cells. We found not only high TKT expression, but also its nuclear localization was a prediction for poor prognosis of HCC patients. We further identified the nuclear localization sequences (NLS) for TKT and demonstrated the NLS mutations decreased the pro-tumor function of TKT independent of the enzyme activity. Cross-linking Co-IP/MS showed that nuclear TKT interacted with kinases and transcriptional coregulators such as EGFR and MAPK3, which are associated with cell activation or stress response processes. EGF treatment significantly increased the viability and proliferation of HCC cells in the enzyme-inactivating mutation TKT-D155A overexpression cells but not in the NLS-D155A double mutant group, which could be blocked by EGFR inhibitor erlotinib treatment.

**Conclusions:**

Our research suggests that in addition to the metabolic manner, TKT can promote the development of HCC in a non-metabolic manner via its nuclear localization and EGFR pathway.

**Electronic supplementary material:**

The online version of this article (10.1186/s13046-019-1131-1) contains supplementary material, which is available to authorized users.

## Background

Liver cancer is the second leading cause of cancer-related deaths worldwide [1]. Hepatocellular carcinoma (HCC), which accounts for 80% of primary liver cancers, is a highly aggressive tumor derived from hepatocytes [2]. As only 40% of patients with hepatocellular carcinoma (HCC) are diagnosed at an early stage [3], the therapeutic options of HCC are often limited. Most HCC patients die of the metastasis and recurrence of HCC even after ablation or resection [4]. It is well known that HCC cells acquire a succession of capabilities for the tumorigenesis and metastatic dissemination [11], however, the molecular mechanisms of these processes remain poorly understood.

The major risk factors for HCC are the presence of cirrhosis and HBV/HCV infection [6]. To survive and develop in the environment of a diseased liver with fibrosis, cirrhosis and inflammation, HCC cells have to adapt to different stresses such as oxidative stress, nutrient starvation, and hypoxia [7–10]. One of the hallmarks of cancer cells is metabolic reprograming [11]. Cancer cells tend to produce ATP by anaerobic glycolysis rather than mitochondrial oxidative phosphorylation, even when the oxygen supply is adequate [12, 13]. Increased glycolysis and pentose phosphate pathway (PPP) regulates the cancer cells to adapt to the large demand for macromolecular precursors for proliferation and to maintain reactive homeostasis against oxidative stress during metastasis progression [5]. In addition to their well-known enzymatic functions, many metabolic enzymes have been shown to have non-metabolic functions in cancer cells. For example, Hexokinase 2 (HKII), Glucose-6-Phosphate Isomerase (PGI), Phosphofructokinase Liver Type (PFK1) and Triosephosphate Isomerase 1 (TPI) have been reported to participate in different cell processes via non-metabolic mechanisms [14]. Furthermore, Glyceraldehyde-3-Phosphate Dehydrogenase (GAPDH) [15–17], Pyruvate Kinase M2 (PKM2) [18], Coenzyme Q7 Hydroxylase (CLK-1) [19] and Aldolase A (ALDO1) [20] have been reported to translocate to the cell nucleus and play direct or indirect roles in transcriptional regulation. These findings provided a novel way to elucidate the non-metabolic effects of the metabolic reprograming in cancer.

Transketolase (TKT) is a key enzyme in the non-oxidative phase of PPP [21]. Three genes have been identified in the human genome encoding TKT isozymes: *TKT*, *TKTL1* and *TKTL2* [22]. TKT was the majority of transketolase not only in human normal organs but also in most tumor tissues while TKTL1 and TKTL2 are mainly expressed in testis [23, 24]. In the present study, we found that *TKT* was associated with HCC metastatic potential. We validated that TKT promoted the proliferation and migration of HCC cells, as well as the viability under oxidative stress in vitro and accelerated the growth and metastasis of HCC cells in vivo.

Interestingly, we found a substantial amount of TKT, distinct from other PPP enzymes, located in the nucleus of HCC cells and tissues. We found not only high TKT expression, but also its nuclear localization was a prediction for poor prognosis of HCC patients. In order to demonstrate the function of nuclear TKT in HCC, we determined the nuclear localization sequence (NLS) of TKT and found that the enzymatic activity of TKT was not affected by its NLS mutation. Therefore, we introduced TKT NLS mutant and TKT enzyme-inactivating mutant in further functional validation. In vitro experiments showed that the NLS mutations decreased the pro-tumor function of TKT independent of its enzymatic activity, implying non-metabolic functions of TKT in the HCC nucleus. Proteomic analysis using cross-linking Co-immunoprecipitation combined with mass spectrometry (cross-linking Co-IP/MS) revealed that nuclear TKT interacted with kinases and transcriptional coregulators such as epidermal growth factor receptor (EGFR) and mitogen-activated protein kinase 3 (MAPK3), which are associated with cell activation or stress response processes. We validated that EGF treatment significantly increased the viability of TKT wild-type cells instead of the NLS mutant group, which could be blocked by EGFR inhibitor erlotinib treatment. These observations uncover that TKT plays an important role in the development of HCC and highlight the function of TKT in both metabolic and non-metabolic pathways.

## Methods

### Materials

High metastatic potential HCC cell lines (HCCLM3, MHCC97H and MHCC97L), and low metastatic potential HCC cell lines (PLC/PRF/5, Huh7, Hep3B and HepG2) [25–27] were purchased from the Liver Cancer Institute, Zhongshan Hospital (Shanghai, China). The information of the HCC cell lines was listed in Additional file 1: Table S1. The HEK293T cell line was purchased from the cell bank of the Chinese Academy of Sciences (Shanghai, China). Nude mice (BALB/c nu/nu) were purchased from Shanghai SLAC Laboratory Animal Co., Ltd. (Shanghai, China) and received humane care according to the “Guide for the Care and Use of Laboratory Animals” published by NIH. Three long-acting siRNA(2′-O-methyl-siRNA) sequences against *TKT* (si-TKT-1: CTGCCGAACTGCTGAAGAA; si-TKT-2: CAGGAGATCTACAGCCAGA; si-TKT-3: GGTAGAAGATAAGGAGTCT) and one negative control were obtained from RiboBio Co., Ltd. (Guangzhou, China). TKT (11039–1-AP), EGFR (18986–1-AP), IPO4 (11679–1-AP), NUP54 (16232–1-AP), ACTB (20536–1-AP), TUBB (10094–1-AP) and LAMB (23498–1-AP) antibodies were purchased from the Proteintech Group, Inc. (Chicago, USA). TKTL1 (GTX109459) and TKTL2 antibodies (GTX118468) were purchased from GeneTex, Inc. (CA, USA). MAPK3(4695) was purchased from CST, Inc. (MA, USA). Erlotinib were purchased from Selleckchem, Inc. (TX, USA). Other chemicals were purchased from Sigma-Aldrich, Inc. (MO, USA).

### Clinical specimens

Two hundred eighty-six cases of HCC tumor samples were collected from patients who underwent surgical resections at the Liver Cancer Institute, Zhongshan Hospital, Fudan University (Shanghai, China) between February 2004 and December 2006. The patients were not treated with any preoperative therapy and received follow-up until May 2008 after surgical treatment. During the follow-up period, the patients were monitored every 2 months. The period ranged from 2 to 28 months, with an average time of 16 months. All the patients provided informed consent approved by the Ethics Committee of Fudan University (Shanghai, China) before enrolling in the present study. The tumor samples were used to construct the tissue microarray (Shanghai Biochip Co., Ltd. Shanghai, China) and stained for TKT. Two pathologists who were blinded to the clinical outcome scored the staining intensity and the extent of the protein expression across the section for each array independently. The staining intensity was classified into three grades as follows: 0 (negative), 1 (weak), 2 (strong). The extent of staining was the percentage of positive tumor cells. The score for each sample was the product of the staining intensity grade multiplied by the positive tumor cell percentage.

### Immunohistochemistry (IHC)

Tissues were fixed in 4% paraformaldehyde, embedded in paraffin and cut into 4-μm sections. After deparaffinization, the sections were microwaved in 0.01 M sodium citrate (pH 6.0) for 10 min for rehydration and antigen retrieval. Appropriate primary antibodies and secondary antibodies were applied to the sections incubating at 4 °C overnight and at room temperature for 1 h, respectively. The DAB horseradish peroxidase color development kit (Beyotime, Haimen, China) was used for immunostaining.

### Cell culture and establishment of stable cell lines

The cell lines were cultured in Dulbecco’s High Glucose Modified Eagle’s Medium (SH30022.01, Thermo Fisher Scientific, MA, USA) supplemented with 10% fetal bovine serum (SS0415, Biochrome, Berlin, Germany) at 37 °C in a humidified incubator containing 5% CO_2_.

To establish stable TKT knockdown cell lines, short hairpin RNA (shRNA) sequences against the TKT sequence (sh-TKT-1: CGCCGAACTGCTGAAGAAAGAA; sh-TKT-2: GCCATCATCTATAACAACAAT) and NC (sh-NC: CCTAAGGTTAAGTCGCCCTCG) were cloned into the pMKO.1 puro retroviral vector. The recombinant vectors were co-transfected into HEK293T cells with GAG-POL and VSVG plasmids using Lipofectamine 2000 (Invitrogen, MA, USA). For the TKT overexpression stable cell lines, TKT cDNA (NP_001128527.1) with Flag-tag at the C-terminus was cloned into pCDH-CMV-MCS-EF1-puro or pCDH-CMV-MCS-EF1-copGFP vector. The recombinant vector was used to package pseudoviral particles with two other packaging plasmids psPAX2 and pMD2.G by co-transfecting into HEK293T cells. The particles were harvested twice after 24 h and 48 h and were used to infect HCC cell lines. Stable pools were selected with 6 μg/mL puromycin (except pCDH-CMV-MCS-EF1-copGFP). In order to identify the NLS of TKT, enhanced green fluorescent protein (eGFP)-tagged TKT sequence truncations and mutants were established as follows: coding sequences for truncated TKT and single point mutants were fused in frame to the eGFP tag sequence in pEGFP-N1 vector. Plasmids were transfected into Huh7 cells using Lipofectamine 2000 for 24 h before fluorescence microscope observation.

### RNA isolation, reverse-transcription, and quantitative real-time PCR (qRT-PCR)

Total RNA was prepared using a TRIzol reagent (Invitrogen, CA, USA) and reverse-transcribed into complementary DNA using PrimeScript® reverse-transcription reagent kit (TAKARA, Dalian, China). Transcriptional level of *TKT*, *TKL1* and *TKTL2* were quantified by real-time quantitative PCR (Additional file 2: Table S2) using SYBR® Premix Ex Taq Kit (TAKARA) on the 7500 Fast Real-Time PCR System (Applied Biosystems, CA, USA). Expression of β-actin was measured as an internal control, and the relative expression levels were evaluated by the *ΔΔ*Ct method. qRT-PCR was performed in triplicate, and standard deviations representing experimental errors were calculated.

### Western blotting

Total cell lysates were prepared from cell lines with RIPA buffer (50 mM Tris-HCl pH 7.4, 150 mM NaCl, 1% Triton X-100, 1% sodium deoxycholate, 0.1% sodium dodecyl sulfate (SDS), 1 mM phenylmethylsulfonyl fluoride (PMSF), protease and phosphatase inhibitor cocktail, Roche, Indiana, USA). Protein concentrations were determined by the bicinchoninic acid (BCA) assay (Beyotime, Haimen, China). Cell lysates supplemented with loading buffer were run on SDS-PAGE gels and then transferred to nitrocellulose membranes (Millipore, Massachusetts, USA). After blocking with 5% Bovine serum albumin (BSA) in tris-buffered saline with Tween 20 (TBST), the membranes were immunoblotted with the appropriate primary and secondary antibodies. ACTB was used as the endogenous control. Protein bands were detected using western blotting Detection Reagents (Bio-Rad Laboratories, California, USA) and ImageQuant™ LAS 3000 (GE Healthcare Life Sciences, Michigan, USA).

### Cell proliferation assay

Cell proliferation was detected with CCK-8 reagent (Dojindo, Kumamoto, Japan). Briefly, the cells were seeded in 96-well culture plates (2 × 10^3^ cells/well). At the indicated time points, 100 μL/well of CCK-8 reaction solution (CCK-8 reagent: mediaum = 1:10) was added to the plates, which were then incubated at 37 °C for 2 h. The absorbance at 450 nm was measured to calculate the number of viable cells in each well.

### Wound healing assay

Cells were seeded in 24-well culture plates (2 × 10^5^ cells/well). After 24 h, when the cells had reached approximately 90% confluence as a monolayer, 200 μL pipette tips were used to create a scratch across the center of the wells. The scratched wells were gently washed and filled with 500 μL DMEM supplemented with 2% FBS. The scratched lines were photographed under a microscope. After culturing the cells for 24–48 h, images were obtained at the same locations and the healing distance was calculated.

### Cell migration assays

Cell migration assays were performed with 24-well transwells (8-μm pore size, Minipore). Cell invasion assays were performed with the same transwells precoated with Matrigel (BD Bioscience, Franklin Lakes, NJ). In total, 1.5 × 10^5^ transfected cells were suspended in serum-free DMEM medium and added to the upper chamber, and 700 μL DMEM with 10% FBS was placed in the lower chamber. After 16 h (migration) or 48 h (invasion) of incubation, cells on the lower surface of membrane were fixed in 4% paraformaldehyde and stained with Giemsa (Sigma Chemical Company, MO, USA). Cells in five microscopic fields were counted and photographed.

### Colony formation experiment

HCCLM3 cell lines were seeded as a concentration of 400 cells/well in the 6 wells plate with 10% FBS DMEM medium. Cells were grown for 14 days to form colonies, which were then stained by crystal violet and counted for statistics.

### In vivo tumor growth and metastasis assays

Stable TKT knockdown and the negative control (NC) HCCLM3 cell lines were injected subcutaneously (5 × 10^6^ cells/animal) into nude mice (6 mice/group) to produce implanted tumors. Tumor volumes were measured every 5 days and calculated as follows: volume = (the larger diameter) × (the smaller diameter) ^2^/2. Mice were sacrificed 4 weeks later. Implanted tumors were dissected and cut into 1 mm^3^ pieces, which were then transplanted into the liver parcel of other nude mice to establish orthotropic implantation models. The mice were sacrificed after 6 weeks. Tumors, livers, and lungs were fixed in 4% paraformaldehyde. Consecutive sections were generated for lung tissues and stained with hematoxylin and eosin. The number of pulmonary metastases was counted. The lung metastases were classified into four grades: Grade I had no more than 20 tumor cells; grade II had 20 to 50 tumor cells; grade III had 50 to 100 tumor cells; grade IV had more than 100 tumor cells.

### TKT activity assay

TKT activity was measured as previously described in multiple studies [28–31] with modifications. All cells were harvested and sonicated in cold lysis buffer (20 mM HEPES, pH 7.5, 10% glycerol, 0.4 mM NaCl, 10 mM EGTA, 5 mM EDTA, 0.4% Triton X-100, 25 mM NaF, 1 mM DTT, and protease inhibitors). The supernatants were collected after centrifugation at 12,000 rpm for 5 min at 4 °C. TKT activity was measured by addition of 40 μL of supernatant to 160 μL of the reaction buffer (50 mM Tris-HCl, pH 7.6; 5 mM MgCl_2_; 0.1 mM thiamine pyrophosphate, TPP; 0.2 mM NADH; 0.2 U/mL triose phosphate isomerase and 0.2 U/mL glycerol dehydrogenase). The reaction mixture was kept at 37 °C for 15 min and 75 μL of a solution containing 25 mM R5P and 25 mM X5P was added. The absorbance at 340 nm was recorded for 30 min.

### Metabolic flux analysis

The metabolic flux analysis was performed as previously described [32]. Briefly, the 1.5 × 10^6^ HCCLM3 cells and sh-TKT-1 cells were cultured in DMEM (Sigma) with 5 mM [1,2-13C_2_]-glucose (Sigma) and 2% dialyzed FBS (HyClone) in 10-cm plates for 12 h. Cell pellets were extracted with methanol/acetonitrile/water (2:2:1), with solvent volumes normalized to a ratio of 1 mL of solvent per 1 mg of cell pellet. The M1/M2 ratio indicates the ratio of glucose cycled through the PPP to glucose going directly through glycolysis. The relative PPP/glycolysis flux was determined with the following formula: relative PPP/glycolysis flux = M1 lactate/(M2 lactate + M1 lactate). Glucose consumption was measured using glucose detection kit(sigma, GAGO20) as manual described.

### Chromatin fractionation

According to the description of O.K. Mirzoeva and J.H. Petrini [33], 10^7^ cells were lysed in 400 μL CSK buffer (10 mM PIPES, pH 6.8; 100 mM NaCl; 300 mM sucrose; 3 mM MgCl2; 1 mM EGTA; 0.5% Triton X-100 supplemented with protein inhibitors) for 10 min. The supernatant collected after low speed centrifugation (5 min, 1500 g, 4 °C) constituted the soluble cytoplasmic fraction, which was subsequently clarified by high-speed centrifugation (10 min, 16,000 g, 4 °C). The low-speed pellets were washed once in CSK buffer and incubated in CSK buffer containing 5 units of RNase-free DNase I (TAKARA, Dalian, China) for 30 min at 37 °C. The DNase pellet was washed once in 1 mL of ice-cold CSK buffer and incubated in ice-cold CSK buffer containing 500 mM NaCl for 10 min at 4 °C. This extract was clarified by centrifugation (10 min, 16,000 g, 4 °C) and pooled with the DNase-treated fraction as the chromatinic protein fraction.

### Cross-linking co-IP/MS

TKT wild-type, TKT K6R along with the empty vector as a negative control were applied to cross-linking Co-IP/MS. For all Huh7 stable cell lines, 10^7^ cells were fixed with 0.5% formaldehyde for 8 min at room temperature (RT) and terminated by addition of 1. 25 M glycine (10×) and incubated for 5 min at RT. Cells were collected and washed 3 times in cold PBS. The pellets were suspended in 900 μL FL buffer [34] (5 mM PIPES pH 8; 85 mM KCl; 0.5% NP-40) with protein inhibitors (Complete Protease Inhibitor Cocktail, EDTA-free, Roche, 11,873,580,001), and sonicated in Bioruptor Plus (Diagenode) at low power (15 s on, 30s off) for 3 cycles and centrifuged at 2000 rpm for 5 min. The nuclear pellets were collected and suspended in 900 μL lysis buffer (50 mM HEPES/KOH, pH 7.5; 500 mM NaCl; 1% Triton X-100; 1 mM EDTA; 0.1% SDS and PI). The cell lysates were sonicated again with Bioruptor Plus with high power mode for 50 cycles (30s on, 30s off) and centrifuged at 12000 rpm for 5 min. The supernatant was collected and precleared with 15 μL Protein G Dynabeads (Thermo Fisher Scientific, MA, USA). Next, 1 μg TKT antibody was added to the lysate and incubated at 4 °C for 4 h, followed by 20 μL Protein G Dynabeads incubation for 1 h. The beads were washed 5 times with lysis buffer and boiled in 40 μL SDS loading buffer for 15 min. The supernatant was separated by SDS-PAGE electrophoresis for one-third of the gel, which was minimally stained with Coomassie brilliant blue, cut into 3 molecular weight ranges and the heavy chain IgG band, and then digested with trypsin [35]. Liquid chromatography-tandem mass spectrometry (LC-MS/MS) analysis was performed with Orbitrap Fusion Lumos equipped with an online Easy-nLC 1000 nano-HPLC system (Thermo Fisher Scientific) as previously described [36, 37].

### Statistical analysis

The experimental data were assessed with SPSS 19.0 (SPSS Inc., Chicago, USA), R 3.4.3 or Microsoft Excel 2016 (Microsoft Inc., Washington, USA). Categorical data were analyzed using the Chi-square tests or Fisher’s exact test. Comparisons between two groups were evaluated using Student’s *t*-test. Comparisons between more than two groups were evaluated using Kruskal-Wallis test (Multiple comparisons). The cumulative survival and recurrence rates were calculated by Kaplan-Meier method, and significant differences were determined using the log-rank test. Kaplan-Meier survival curves were constructed based on cBioPortal database (TCGA-Liver-Cancer) at mRNA level and tissue microarray of 286 HCC samples at protein level [38, 39]. HCC patients were stratified according to TKT expression (high and low). The partition was optimized by maximizing risk groups algorithm. Briefly, the samples were ordered by TKT level, each of them was used as split point for high and low groups and a log-rank test is performed along all values of the arranged groups. The final split point was chosen where the *p*-value is minimum [40, 41].

## Results

### Expression of TKT is positively correlated with HCC metastatic potential and poor prognosis

**Fig. 1:**
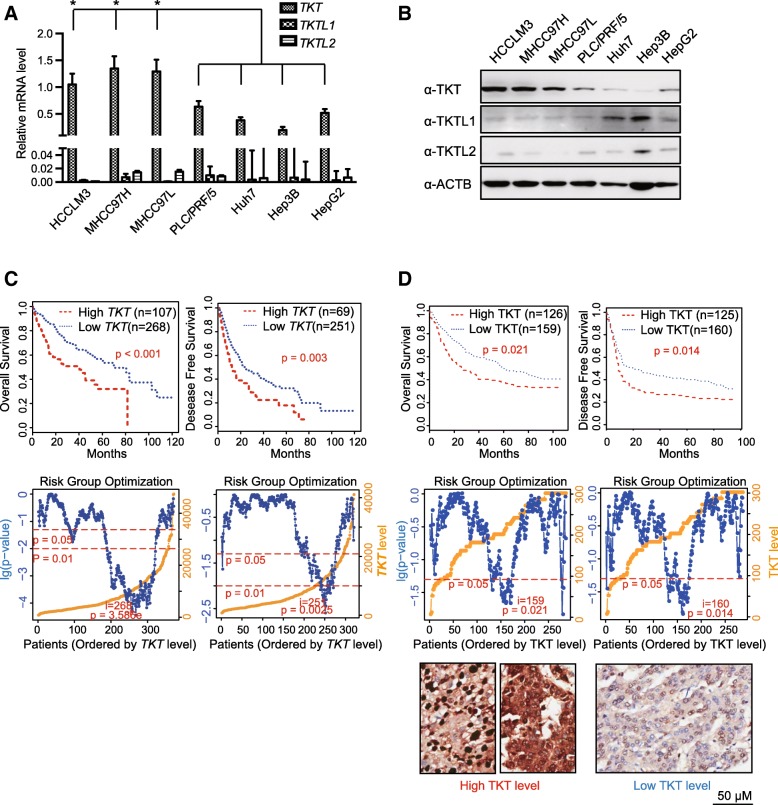
mRNA and Protein Levels of TKT in HCC Cell Lines and HCC Tumor Samples. **a** The mRNA levels of *TKT/TKTL1/TKTL2* in each of the metastatic HCC cells (HCCLM3, MHCC97H and MHCC97L) were compared to those in any of the non-metastatic cells (PLC/PRE/5, Huh7, Hep3B and HepG2) by real-time qPCR. The *TKT* level in each of the metastatic cells was significantly higher than any of the non-metastatic cells (*: *p* < 0.05). *TKTL1* and *TKTL2* were not detected in HCC cells. **b** Protein levels of TKT/TKTL1/TKTL2 in metastatic and non-metastatic HCC cells were validated by western blot analysis. **c** The association of *TKT* mRNA level with overall survival (OS) and disease-free survival (DFS) of HCC patients in the cBioPortal database (TCGA-Liver-Cancer) by Kaplan–Meier analysis (*p* value from log-rank test). Left: OS data set; right: DFS data set. The partition was optimized by maximizing the risk groups algorithm. The split point was chosen where the minimum *p* value was obtained [39]. **d** The association of TKT protein level with OS and DFS of HCC patients (*n* = 285, Kaplan–Meier analysis, *p* value from log-rank test). Left: OS data set; right: DFS data set. The partition was determined by the median score of IHC in the tissue microarray. Representative immunostaining of TKT with high and low level are shown in the bottom panels. The partition was optimized by maximizing the risk groups algorithm. The split point was chosen where the minimum *p* value was obtained [39]

To examine the relationship between TKT encoding genes (*TKT*, *TKTL1*, *TKTL2*) and the metastatic potential of HCC cells, the mRNA and protein level of the three genes were assessed by real-time quantitative PCR and western blotting respectively. The mRNA level of *TKT* in each of the metastatic HCC cell lines, including HCCLM3, MHCC97H and MHCC97L, was significantly higher than in any of the non-metastatic cells, including PLC/PRE/5, Huh7, Hep3B and HepG2. The expression of *TKTL1* and *TKTL2* was negligible compared with *TKT* in all the HCC cell lines (Fig. 1a). Similarly, the protein level of TKT also showed a positive correlation with the HCC cell metastatic potential, while the expression levels of TKTL1 and TKTL2 were undetected in most metastatic HCC cell lines (Fig. 1b). We further compared the expression level of *TKT*, *TKTL1* and *TKTL2* in HCC tissues based on TCGA database [40]. We found *TKTL1* and *TKTL2* were undetected in most HCC tissues and *TKT* was significantly higher than the other two isozymes (Additional file 3: Figure S1 A).

We analyzed the correlation between the TKT expression and both overall survival (OS) and disease-free survival (DFS) of HCC patients. We found high TKT expression was significantly correlated with characteristics of a poor prognosis, such as a high TNM stage (*P* = 0.013), microvascular invasion (*P* < 0.001) and loss of tumor encapsulation (*P* = 0.028) (Table 1). Kaplan-Meier survival analysis showed that high TKT expression at both mRNA and protein levels was significantly correlated with shorter OS and DFS time of the patients (Fig.1c-d), suggesting that high TKT level indicates poor prognosis in HCC patients. These results suggest that TKT may contribute to HCC progression and metastasis.

**Table 1:** Correlation between TKT level and clinicopathological characteristics of 286 HCC patients

Clinicopathological variables		TKT level (High vs. Low)				*p* Value
		High level		Low level		
		Mean	Count	Mean	Count	
Age		51		53		
Sex	Female		20		30	0.337
	Male		112		124	
Cirrrhosis	Absent		99		118	0.759
	Present		32		35	
Maximal tumor size	< 5 cm		98		117	0.735
	≥ 5 cm		34		37	
Tumor number	Single		123		145	0.735
	Multiple		9		9	
HBV infection	no		4		10	0.176
	yes		128		144	
Anti_HCV	negtive		132		153	0.354
	positive		0		1	
TB	< 20 μM/L		104		106	0.057
	≥ 20 μM/L		28		48	
AFP	< 20 ng/mL		46		53	0.939
	≥ 20 ng/ml		86		101	
Microvascular invasion	absent		65		113	< 0.001*
	present		67		41	
Loss of Tumor encapsulation	Complete		60		90	0.028*
	Not complete		72		64	
Child-pugh score	A Class		20		31	0.233
	B or C Class		109		116	
TNM stage	I-II		118		149	0.013*
	III-IV		14		5	

Results are based on nonempty rows and columns in each innermost subtable

^*^The Chi-square statistic is significant at the 0.05 level

### TKT promotes HCC growth and metastasis in vitro and in vivo

**Fig. 2:**
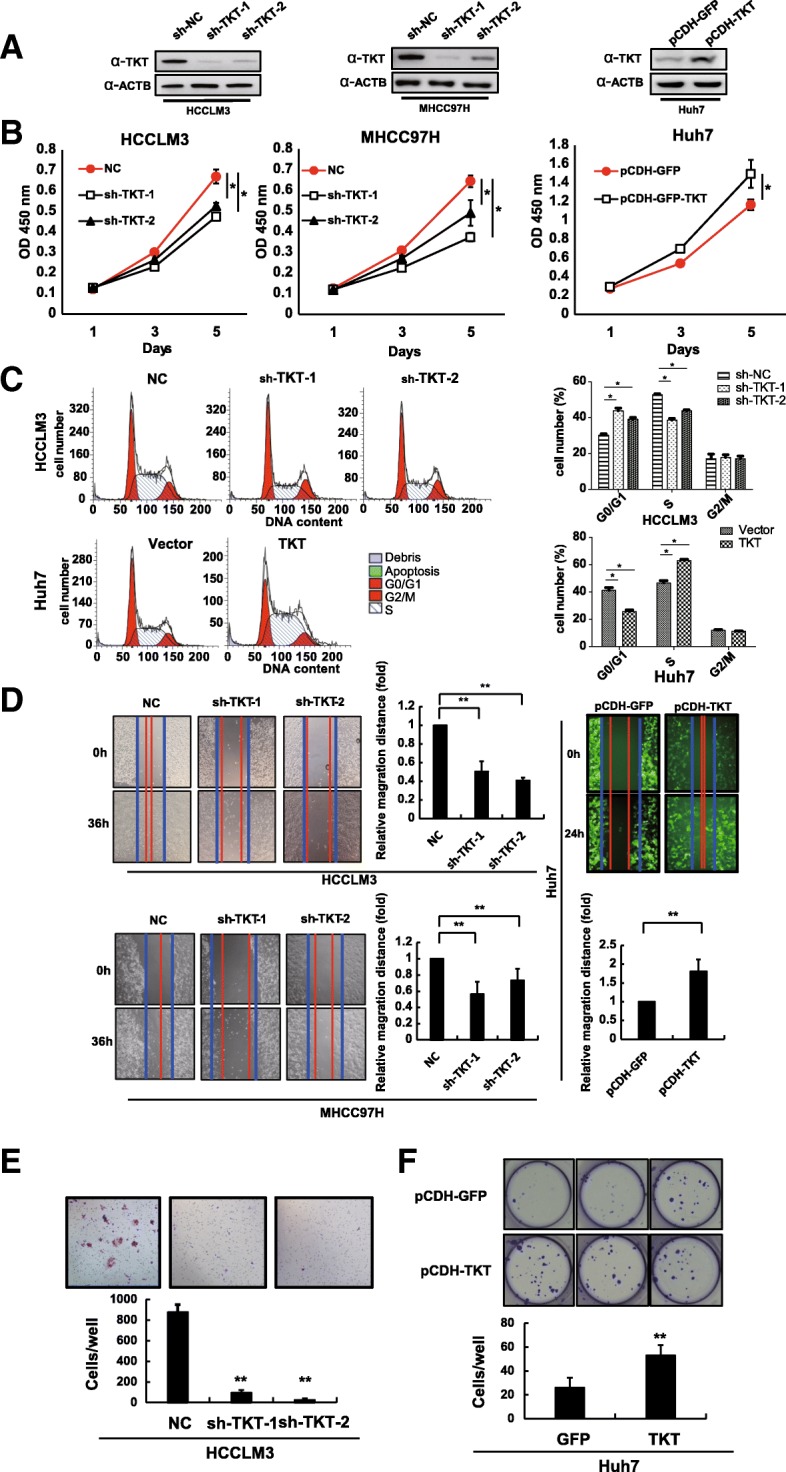
TKT Enhanced the Proliferation, Migration, Invasion and Colony Formation of HCC Cells in vitro*.* **a** Protein levels of TKT in different HCC stable cell lines were evaluated by western blot. **b** Proliferation assays showed that knocking down TKT suppressed and overexpressing TKT enhanced the proliferation of HCC cells. **c** Cell cycle assay showed that TKT promoted the DNA synthesis and the transition from G0/G1 to S phase in HCC cells. **d** Wound-healing assays showed that knocking down TKT suppressed and overexpressing TKT enhanced the migration of HCC cells. **e** Transwell assay with Matrigel showing that knocking down TKT suppressed invasion of HCC cells. **f** Overexpressing TKT enhanced the colony formation of HCC cells (*: *p* < 0.05; **: *p* < 0.01)

To verify the roles of TKT in HCC tumorigenesis and metastatic progression, multiple in vitro experiments were performed. Knockdown stable cell lines were established in HCCLM3 and MHCC97H cell lines using two efficient short hairpin RNA (sh-RNA) sequences, and TKT overexpressing stable cell lines were prepared using Huh7. TKT expression in all the stable cell lines above was validated by western blotting (Fig. 2a). We also detected the TKTL1 and TKTL2 protein levels in TKT knocking-down and overexpressing HCC cell lines to determine whether there was any compensatory alteration of the other two TKT isozymes which might influence the following experiments. Western blot results showed that the level of TKTL1 and TKTL2 were not affected by knocking-down or overexpressing TKT (Additional file 3: Figure S1B). After knocking down TKT, HCC cell proliferation was suppressed (HCCLM3 and MHCC97H), whereas over-expression of TKT promoted HCC cell proliferation (Fig. 2b). TKT mediates a series of reversible reactions in PPP synthesizing 5-carbon sugars, providing precursors for nucleotides. It would be reasonable that TKT could promote the DNA synthesis and the transition from G0/G1 to S phase by offering sufficient precursors of DNA. The cell cycle assay verified that the percentage of cells in G0/G1 phase was down-regulated and the percentage of cells in S phase was significantly up-regulated in TKT over-expressing Huh7 cells. Meantime, HCCLM3 cells were arrested in G0/G1 phase when TKT was knocked down resulting in decreased percentage of S phase (Fig. 2c).

The wound healing assay revealed that down-regulation of TKT decreased the migration of the HCC cells HCCLM3 and MHCC97H, whereas up-regulation of TKT significantly increased this ability in the Huh7 cell line (Fig. 2d). Furthermore, using the Matrigel invasion assay, we found that the invasion ability was suppressed if TKT was down-regulated in the HCCLM3 cell line (Fig. 2e). In the clonogenic assay, up-regulation of TKT promoted the clonogenic ability of Huh7 cells, showing that the TKT level was positively related to the colony formation ability of HCC cells (Fig. 2f). In summary, these results suggest that TKT could enhance the proliferation, migration, invasion and colony formation ability of HCC cells in vitro.

**Fig. 3:**
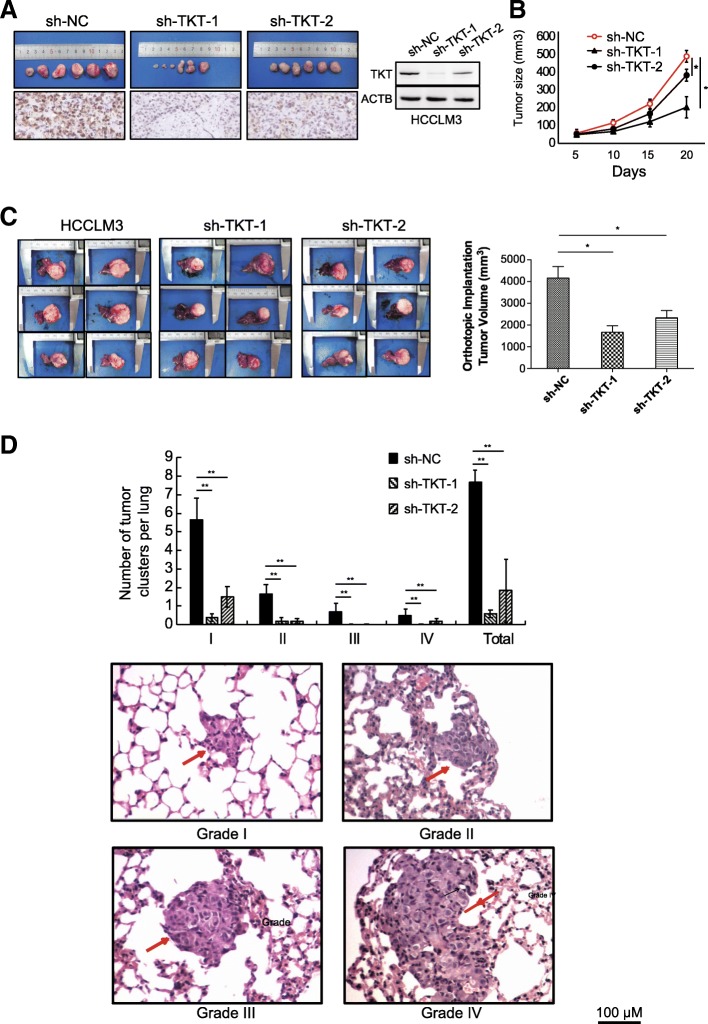
TKT Promotes HCC Cell Growth, Proliferation and Distant Metastasis in vivo. **a** Knocking down TKT inhibited the subcutaneous growth of HCCLM3 cells in nude mice. **b** The growth curves of subcutaneous tumors in nude mice treated by negative control and TKT knockdown HCCLM3 cells. **c** Implanted tumor burden in mouse livers (6 weeks after orthotopic xenograft transplantation of subcutaneous tumors into mouse livers). **d** Upper: the number of lung metastatic foci in each group was calculated. Classification of metastatic foci was based on the cell number in the metastatic lesion. Lower: representative lung tissue sections from each group were shown (hematoxylin & eosin stain, scale bar, 100 μm). Red arrows indicate lung metastatic tumors (*: *p* < 0.05; **: *p* < 0.01)

We further examined the effect of TKT on the tumorigenesis and pulmonary metastasis by establishing an orthotropic liver tumor model in nude mice. Two TKT-knockdown stable cell lines with different TKT expression patterns (sh-TKT-1 with the lowest TKT expression, sh-TKT-2 with the second lowest TKT expression) and mock control cells (sh-NC) established from HCCLM3 cells were used to form subcutaneous tumors in nude mice. The results showed that TKT expression was positively correlated with subcutaneous tumor size (Fig. 3a-b). The subcutaneous tumor tissues were then orthotopically implanted into nude mouse livers. The growth of implant tumor of TKT knock-down groups were significantly inhibited compared to the NC group (Fig. 3c). Distant metastatic nodes in the lung of the TKT knockdown groups were significantly reduced compared with the control groups, and a positive correlation was observed between the number of different grades of metastatic nodes and the TKT level (Fig. 3d). Together, these results reveal an effect of increased TKT on the tumorigenesis and metastasis of HCC in vitro and in vivo.

### TKT sustains the ratio of NADPH/NADP and GSH/GSSG and enhances the viability of HCC cells against oxidative stress in a metabolic manner

**Fig. 4:**
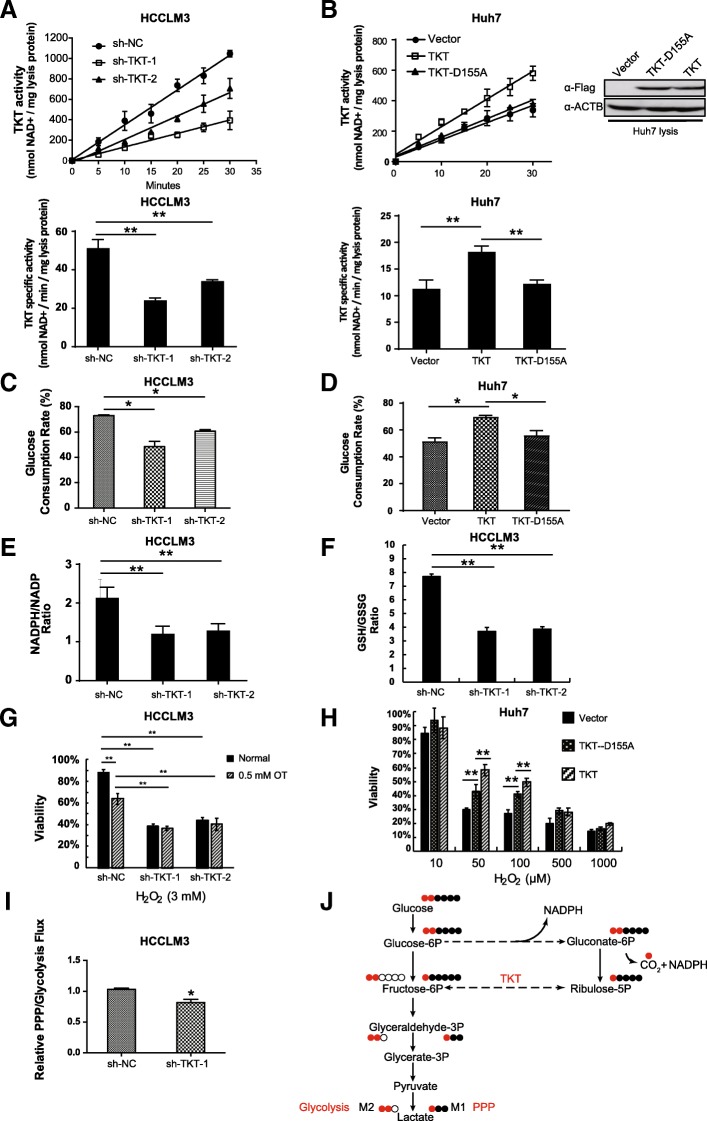
TKT sustains the ratios of NADPH/NADP and GSH/GSSG and enhances viability in response to oxidative stress in HCC cells. **a** TKT activity in TKT knocking-down cell lines (HCCLM3). **b** TKT activity in TKT and TKT enzyme-inactivating mutant overexpression cell lines (Huh7). **c** The glucose consumption rate of TKT knocking-down cell lines (HCCLM3). **d** The glucose consumption rate of TKT and TKT enzyme-inactivating mutant overexpression cell lines (Huh7). **e** NADPH/NADP ratio in TKT knockdown cell lines (HCCLM3). **f** GSH/GSSG ratio in TKT knockdown cell lines (HCCLM3). **g** TKT knockdown inhibited the viability of HCCLM3 cells under oxidative stress by treatment with 3 mM H_2_O_2_, even when TKT enzymatic activity was inhibited by 0.5 mM Oxythiamine (OT). **h** TKT overexpression (not only wild-type TKT, but also the TKT-D155A enzyme-inactivating mutant) increased the viability of Huh7 cells under oxidative stress by treatment with H_2_O_2_. **i** Relative PPP/Glycolysis Flux. **j** Overview of isotopomer formation through carbon flux distribution of [1,2-^13^C_2_]-glucose. The figure showed lactate labeling when1,2-^13^C_2_ glucose is metabolized through glycolysis directly or fed back through the PPP. Red circles correspond to ^13^C-labeled carbons, and black circles correspond to unlabeled ^12^C carbons (*: *p* < 0.05; **: *p* < 0.01)

TKT is the key enzyme of the non-oxidative phase of PPP and mediates a series of reversible reactions connecting carbon flux of PPP to glycolysis. We verified that knocking-down TKT (Fig. 2a) significantly decreased the TKT activity (Fig. 4a) and the glucose consumption of HCC cells (Fig. 4c). PPP generates NAPDH and synthesizes 5-carbon sugars, providing major reducing power and precursors for nucleotides. We found that NADPH/NADP and GSH/GSSG ratios, which represent the reducing power in the tumor cells, were down-regulated in TKT knock-down HCCLM3 cells (Fig. 4e&f). We then examined the effect of TKT on HCC cells under oxidative stress conditions. Down-regulation of TKT significantly inhibited the viability of HCCLM3 cells with the treatment of H_2_O_2_. Moreover, the viability of HCC cells under oxidative stress was also suppressed after treatment with TKT enzyme inhibitor oxythiamine (OT), suggesting that the enzyme activity of TKT is crucial for HCC cells (Fig. 4g). To further investigate the metabolic mechanisms of TKT in HCC cells, we introduced TKT with dominant-negative mutation to construct overexpression stable cell lines in further analysis. It has been reported that aspartate 155 of TKT is essential for the enzyme activity [42]. We overexpressed the enzyme-inactivating mutant TKT-D155A in Huh7 cell line and found D155A mutation would abolish the TKT enzymatic activity (Fig. 4b). Similarly, the glucose uptake rate (Fig. 4d) and the viability (Fig. 4h) of wild-type TKT overexpression group was significantly higher than the control and the TKT-D155A groups.

In order to further demonstrate that TKT promotes the HCC viability in a metabolic manner, we applied a ^13^C labeling method [32, 43] tracing ^13^C labels from 1,2-^13^C_2_ glucose by liquid chromatography/mass spectrometry (LC/MS) and analyzed the carbon fluxes of the PPP and glycolysis. PPP branches from glycolysis, converts glucose-6-phosphate into five-carbon sugars for the ribose synthesis and generates the majority of NADPH as the essential reductant in cancer cells [32]. The 1,2-^13^C_2_ glucose, which was metabolized directly through glycolysis, would produce lactate containing two ^13^C labels. Meanwhile, when the labeled glucose was metabolized through the PPP, the ^13^C label on the first position of glucose, would be removed by oxidative decarboxylation reaction, producing 5 carbon intermediate metabolites containing one ^13^C label. These metabolites would then be reinserted into glycolysis via the non-oxidative reactions of the PPP mediated by enzymes including TKT, resulting in the production of lactate containing one ^13^C label (Fig. 4j). The ratio of lactate with one ^13^C label (M1) to that with two ^13^C labels (M2) would represent the ratio between PPP flux and the direct glycolysis flux. We found knocking-down TKT would reduce the PPP flux into glycolysis (Fig. 4i). Considering the overall glucose uptake was also inhibited in TKT-knockdown cells, it could be concluded that down-regulation of TKT decreased the carbon flux through PPP and the production of NAPDH.

These results indicate that the enzyme activity of TKT contributes to the regulation of the production of NAPDH that counteracts oxidative stress in HCC cells.

### TKT translocation to the nucleus and interaction with the chromatin depends on a nuclear location signal

Interestingly, we found that even after the treatment of TKT inhibitor OT, the viability of sh-NC group was still higher than that of TKT knocking-down cells (Fig. 4g). Similarly, although the viability of the TKT-D155A Huh7 cells was significantly lower than the wild-type TKT overexpressing cells, it was still higher than the control group (Fig. 4h), suggesting that there might be a non-enzymatic mechanism of TKT to promote HCC cells viability.

**Fig. 5:**
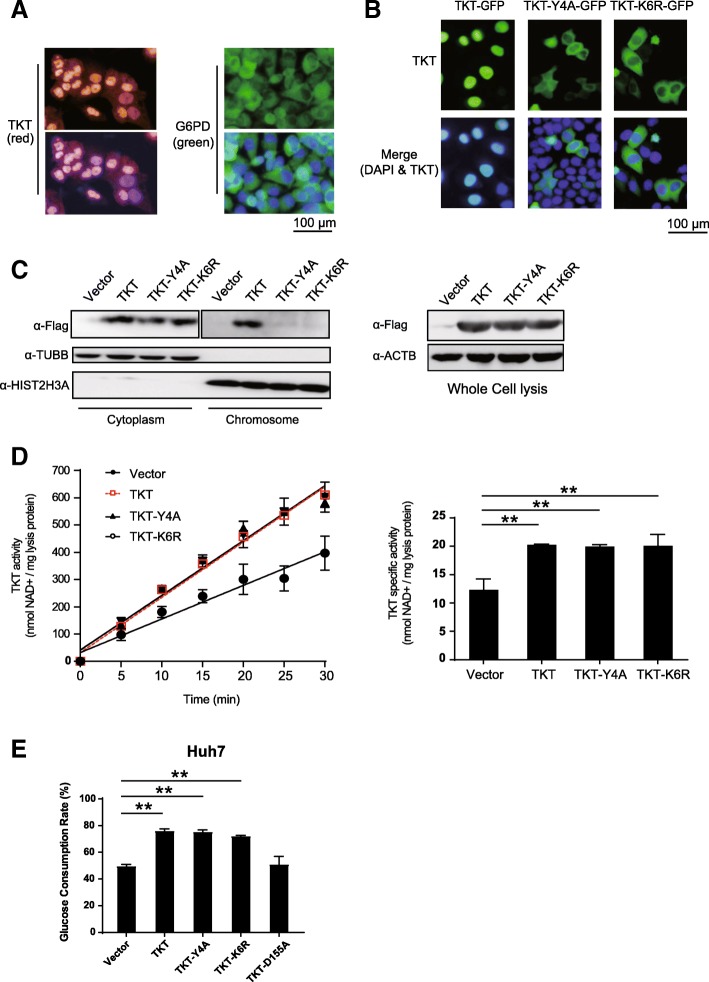
TKT translocates to the nucleus and interacts with chromatin in HCC cells via a specific NLS. **a** Immunofluorescence staining of TKT and G6PD in HCCLM3 cells. Unlike the other enzymes of PPP, TKT showed a significant nuclear localization. **b** Subcellular localization of GFP-tagged TKT, TKT-Y4A and TKT-K6R mutants in Huh7 (green: TKT; blue: nucleus). **c** Cytoplasmic and chromatic protein was extracted from stable Huh7 cells with Flag-tagged TKT wild-type, TKT-Y4A, TKT-K6R overexpression vectors, and TKT levels were examined. **d** Enzymatic activity of TKT wild-type, TKT-Y4A and TKT-K6R in the overexpressing Huh7 cells. **e** Glucose consumption of TKT wild-type, TKT-Y4A, TKT-K6R and TKT-D155A (*: *p* < 0.05; **: *p* < 0.01)

Glycolysis and PPP mainly occur in the cytoplasm [44]. TKT enzymatic activity is reported to occur in the cytoplasmic organoids, such as peroxisomes and the endoplasmic reticulum, in human liver cells [45]. Thiamine pyrophosphate (TPP), the cofactor of TKT, has also been reported to be localized in cytosolic pools, mitochondria and peroxisomes [46]. However, unlike other key enzymes of PPP, TKT immunostaining was observed in the nucleus of HCC cells (Fig. 5a) and HCC tissue samples (Fig. 1d). To validate these results, we overexpressed the GFP-tagged TKT fusion protein in Huh7 cells. Its subcellular location was consistent with the immunostaining results (Fig. 5b, Additional file 3: Figure S2A). These results suggest that the nuclear localization of TKT might promote HCC development in a potential non-metabolic manner.

To better understand the potential non-metabolic functions of TKT, we located the NLSs of TKT. First we overexpressed two GFP-tagged TKT truncations covering 2/3 of the full length from the N or C terminus, respectively. Neither truncated protein localized to the nucleus (Additional file 4: Figure S2 A), suggesting the presence of an NLS in both the N and C termini of TKT. We then established a series of truncations and mutants, and found that tyrosine 4 and lysine 6 in the N-terminus as well as the segment of hydrophobic amino acids consisting of aspartate-alanine-isoleucine-alanine (DAIA) from 610 to 614 in the C-terminus independently mediated the nuclear localization of TKT. However, any mutations in DAIA would destabilize TKT, while the Y4A and K6R mutations would abolish the nuclear localization of TKT without leading to a degradation of TKT (Additional file 1: Figure S2 B-C, Fig. 5b). We subsequently established stable cell lines in huh7 cells using Flag-tagged TKT wild-type, TKT-Y4A, TKT-K6R overexpression vectors. The cytoplasm and chromatin fractions of these TKT mutated cells along with wild-type and mock control cells were extracted. Only wild-type TKT could be detected in the chromatin fraction (Fig. 5c). Moreover, we compared the enzymatic activity and glucose consumption of TKT wild-type, TKT-Y4A and TKT-K6R overexpressing cells. Unlike the enzyme-inactivating mutant TKT-D155A, the NLS mutation did not affect the activity and glucose consumption of the overexpressing cell lines (Fig. 5d-e). These results demonstrate that the Y4 and K6 in the N-terminus could mediate TKT translocation to the nucleus and interaction with chromatin without altering the enzyme activity.

### Nuclear TKT promotes the proliferation, viability, and migration of HCC cells in a non-metabolic manner and predicts a poor prognosis of HCC patients

**Fig. 6:**
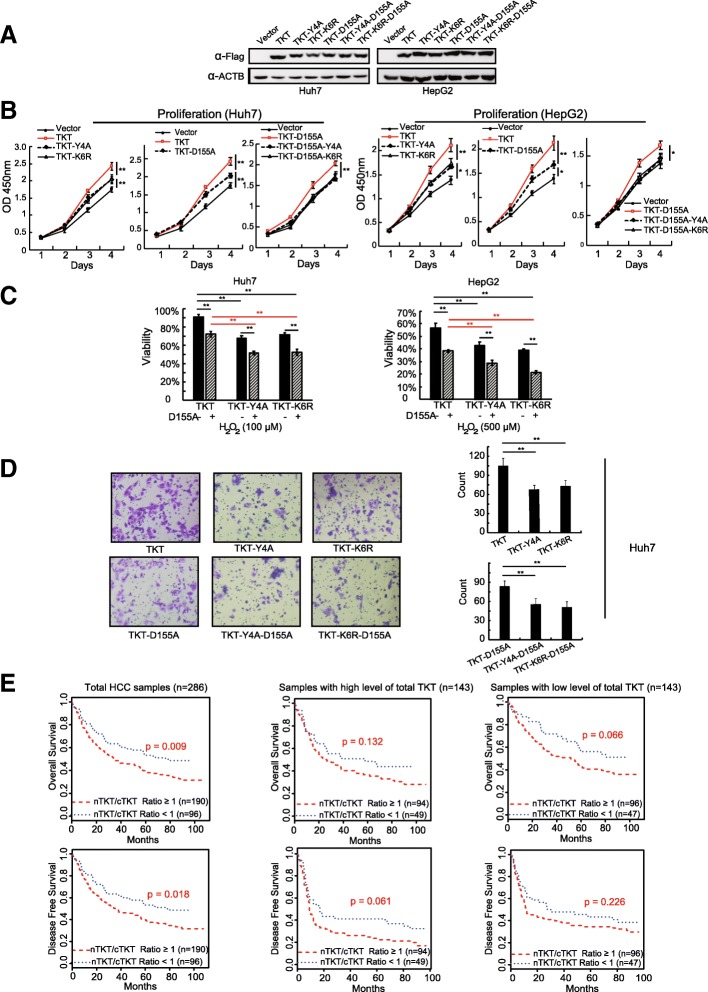
Nuclear TKT promotes the proliferation, viability, and migration of HCC cells in a non-metabolic manner and predicts a poor prognosis of HCC patients. **a** TKT overexpressing stable cell lines, including TKT wild type, TKT-Y4A, TKT-K6R, TKT-D155A, TKT-D155A-Y4A, TKT-D155A-K6R in Huh7 and HepG2 cells were established and validated by western blot. **b** Proliferation rates of TKT wild type, TKT-Y4A and TKT-K6R with or without the D155A dominant-negative mutation in Huh7 and HepG2 cells. **c** Viability of TKT wild type, TKT-Y4A and TKT-K6R with or without the D155A dominant-negative mutation in Huh7 and HepG2 cells under oxidative stress. Cells were treated with H_2_O_2_ for 6 h. **d** Migration of TKT wild type, TKT-Y4A and TKT-K6R with or without the D155A dominant-negative mutation in Huh7 and HepG2 cells. **e** The association of nuclear TKT level with OS and DFS of HCC patients (*n* = 286, Kaplan–Meier analysis, *p* value from log-rank test). Upper: OS data set; lower: DFS data set. nTKT/cTKT, the nuclear TKT to the cytoplasmic TKT ratio. Low, nTKT/cTKT ratio < 1, *n* = 96. High, nTKT/cTKT ratio ≥ 1, *n* = 190. Nuclear and cytoplasmic TKT levels were scored by IHC (*: *p* < 0.05; **: *p* < 0.01)

To further validate the non-metabolic functions of nuclear TKT in HCC cells, a series of TKT overexpressing stable cell lines, including TKT-Y4A (or TKT-K6R), TKT-D155A and TKT-D155A-Y4A (or TKT-D155A-K6R) in Huh7 and HepG2 cells were established (Fig. 6a). The proliferation rates of the TKT-Y4A, TKT-K6R and the enzyme-inactivating mutant TKT-D155A (TKT without metabolic enzyme activity) cell lines were significantly lower than the TKT wild-type group, respectively (Fig. 6b). TKT wild type overexpression promoted the transition from G0/G1 to S phase, while the NLS mutant receded this progress, indicating nuclear TKT involved in the regulating of cell cycle (Additional file 5: Figure S3A-B). Although TKT enzyme-inactivating mutant D155A reduce the function of regulating cell cycle, the expression of TKT-D155A still increase the percentage of S phase, suggesting there would be a non-metabolic mechanism of TKT to regulate cell cycle (Additional file 5: Figure S3A,C). We also determined the glucose consumption of TKT and TKT with NLS mutations. The results showed that the NLS mutation did not affect the glucose uptake compared with TKT wide-type (Additional file 5: Figure S3 E). These results indicated that the nuclear location of TKT would contribute to the cell proliferation independent of TKT enzymatic activity. We then compared the proliferation rates of TKT-D155A with or without the NLS mutated cell lines (TKT-D155A, TKT-D155A-Y4A and TKT-D155A-K6R). The proliferation rates of TKT with Y4A-D155A or K6R-D155A double-mutation showed no difference with vector control, which was lower than TKT-D155A (Fig. 6b). Meantime, we noticed that the proliferation rate of Y4A/K6R mutation was still significantly higher than vector control and only D155A and Y4A/K6R double-mutation could totally abolish the pro-proliferation function of TKT. Similarly, TKT-K6R-D155A double mutation showed no significant function of regulating cell cycle (Additional file 5: Figure S3 D), suggesting the nuclear location and enzyme activity of TKT promote the DNA synthesis and the transition from G0/G1 to S phase in HCC cells independently.

Furthermore, we examined the viability of TKT, TKT-Y4A and TKT-K6R with or without the D155A mutation in Huh7 and HepG2 cells under oxidative stress. The viability of TKT wild-type overexpressing cell line was significantly higher than TKT NLS mutant overexpressing cells, even in the presence of enzyme-inactivating mutation D155A (Fig. 6c). The same trends were also observed in cell migration assays (Fig. 6d).

In summary, TKT could promote HCC cells proliferation, viability and migration by its nuclear localization and enzymatic function independently.

Next, we analyzed the relationship between the nuclear distribution of TKT and the overall or disease-free survival in HCC patients. To avoid the influence from TKT overall expression condition, the HCC patients were separated into high and low TKT groups by TKT expression level in whole cells (total TKT). Nuclear and cytoplasmic TKT levels were then scored respectively. The high and low TKT groups were further divided by the ratio of nuclear TKT to cytoplasmic TKT (relative level of nuclear TKT; high: ratio ≥ 1; low: ratio < 1). Correlation analysis indicated that either in high total TKT group or low TKT group, a relatively high level of nuclear TKT was significantly correlated with clinical pathological variables predicting impaired liver function and a poor prognosis of surgical treatment of HCC (Table 2). Kaplan-Meier survival analysis showed that patients with relatively high nuclear TKT had a shorter disease-free survival (*P* = 0.018) and a shorter overall survival (*P* = 0.009) than that of the relatively low nuclear TKT group. The same trends were also observed in both high total TKT and low total TKT sub-groups (Fig. 6e). Univariate and multivariate analysis of OS and DFS verified that the relative level of nuclear TKT was an independent and significant risk factor for a poor survival rate in HCC patients (Table 3). These results indicate that nuclear TKT could enhance the proliferation, viability, and migration of HCC cells via a non-metabolic mechanism and high TKT in the nucleus predicts a poor prognosis of HCC patients.

**Table 2:** Correlation between ratio of nucleus to cytoplasm TKT level and clinicopathological characteristics of 286 HCC patients

Clinicopathological variables		Ratio of nucleus to cytoplasm (≥ 1versus < 1)										
		High total TKT level (*n* = 143)						Low total TKT level (n = 143)				
		≥1		< 1		*p* Value		≥1		< 1		*p* Value
		Mean	Count	Mean	Count			Mean	Count	Mean	Count	
Age		52		51				53		53		
Sex	Female		13		10	0.490			18		9	0.749
	Male		77		43				81		35	
Cirrrhosis	Absent		70		36	0.160			77		34	0.862
	Present		19		17				21		10	
Maximal tumor size	< 5 cm		59		45	0.012*			75		33	0.923
	≥ 5 cm		31		8				24		11	
Tumor number	Single		83		50	0.632			92		43	0.249
	Multiple		7		3				7		1	
HBV infection	no		3		2	0.890			7		3	0.956
	yes		87		51	.			92		41	
Anti_HCV	negtive		90		53	.			98		44	0.503
	positive		0		0				1		0	
TB	< 20 μM/L		73		38	0.190			70		30	0.761
	≥ 20 μM/L		17		15				29		14	
AFP	< 20 ng/mL		29		19	0.660			30		17	0.328
	≥ 20 ng/ml		61		34				69		27	
Microvascular invasion	absent		44		28	0.650			70		33	0.598
	present		46		25				29		11	
Loss of Tumor encapsulation	Complete		38		27	0.310			54		28	0.310
	Not complete		52		26				45		16	
Child-pugh score	A Class		10		12	0.048*			14		14	0.010*
	B or C Class		79		38				81		27	
TNM stage	I-II		78		50	0.150			96		43	0.800
	III-IV		12		3				3		1	

Results are based on nonempty rows and columns in each innermost subtable

*The Chi-square statistic is significant at the 0.05 level

**Table 3:** Univariate and multivariate analysis of factors associated with disease-free survival and overall survival in 286 HCC patients

Variables	Overall survival						Disease free survival					
	Univariate analysis			Multivariate analysis			Univariate analysis			Multivariate analysis		
	*p* value	HR	95% CI	*p* value	HR	95% CI	*p* value	HR	95% CI	*p* value	HR	95% CI
Age	0.582	1.004	0.991–1.017				0.740	1.002	0.990–1.014			
Sex (female versus male)	0.795	1.054	0.710–1.563				0.221	1.247	0.876–1.777			
Cirrrhosis (absent versus present)	0.524	0.892	0.627–1.268				0.312	0.845	0.611–1.170			
Maximal tumor size (< 5 cm versus ≥5 cm)	0.046*	**0.710**	**0.507-0.994**	0.208	0.790	0.548–1.140	0.118	0.777	0.566–1.066			
Tumor number (single versus multiple)	0.182	0.680	0.386–1.198				0.895	0.963	0.549–1.690			
HBsAg (negative versus positive)	0.014*	**0.448**	**0.236-0.850**	0.170	0.532	0.216–1.311	0.017*	**0.337**	**0.139-0.821**	0.048*	**0.406**	**0.166-0.994**
Anti_HCV (negative versus positive)	0.507	0.049	0.000–359.572				0.452	20.319	0.008–52,097.066			
TBIL (< 20 μM/L versus ≥20 μM/L)	0.037*	**0.704**	**0.507-0.978**	0.011*	**0.644**	**0.459-0.904**	0.081	0.760	0.559–1.034			
AFP (< 20 ng/mL versus ≥20 ng/mL)	0.018*	**0.673**	**0.484-0.935**	0.176	0.788	0.559–1.113	0.013*	**0.685**	**0.508-0.923**	0.074	0.756	0.556–1.027
Vascular invasion (absent versus present)	0.002*	**0.611**	**0.450-0.829**	0.183	0.788	0.555–1.119	0.001*	**0.630**	**0.475-0.836**	0.135	0.789	0.579–1.077
Tumor encapsulation (complete versus not)	0.035*	**0.722**	**0.534-0.977**	0.158	0.798	0.584–1.091	0.028*	**0.731**	**0.553-0.966**	0.229	0.839	0.630–1.117
Child-pugh score (A versus B and C)	0.103	0.709	0.470–1.072				0.121	0.744	0.511–1.082			
TNM stage(I-II versus III-IV)	< 0.001*	**0.322**	**0.192-0.541**	0.007*	**0.466**	**0.269-0.808**	0.003*	**0.461**	**0.276-0.770**	0.132	0.661	0.386–1.132
Ratio of nucleus to cytoplasm(≥ 1versus < 1)	0.012*	**1.543**	**1.102-2.161**	0.024*	**1.485**	**1.053-2.093**	0.023*	**1.424**	**1.050-1.932**	0.042*	**1.38**	**1.011-1.885**
TKT level (High versus Low)	0.023*	**1.419**	**1.049-1.920**	0.090	1.320	0.957–1.821	0.024*	**1.378**	**1.043-1.821**	0.209	1.209	0.899–1.627

*The statistic is significant at the 0.05 level

### Nuclear TKT interacts with transcription factors or signaling pathway molecules and may regulate the stress response and progression of cell activation

The localization of TKT in the chromatin-associated protein fractions in HCC cells (Fig. 5c) indicates that it might be involved in transcriptional regulatory processes via a non-metabolic mechanism. It was important to investigate the interaction proteins of TKT in the nucleus. However, because of the low abundance of transcription factors (TFs) and co-regulators (TCs), key transcriptional regulatory proteins are frequently underrepresented in protein level investigation [47]. Moreover, some of the interactions between TFs, TCs and kinases are weak or transient. Therefore, we used cross-linking Co-IP/MS to assess the proteins that interact with nuclear TKT (Additional file 6: Figure S4 A). Equally overexpressed TKT wild type and TKT NLS mutation (K6R) stable cell lines along with the empty vector control group (Additional file 6: Figure S4 B) were crosslinked by formaldehyde respectively. To avoid contamination with cytoplasmic TKT, we extracted nuclear fractions after the cross-linking (Additional file 6: Figure S4 C). It was verified that only wild type TKT could be detected in nucleus fractions (Additional file 6: Figure S4 D). Due to some steric hindrance effect under the crosslinking condition, FLAG-tag on overexpressed TKT could not be detected by FLAG antibody. As a result, TKT antibody was used to pull down the fixed target protein complexes after crosslinking (Additional file 6: Figure S4 E).

**Fig. 7:**
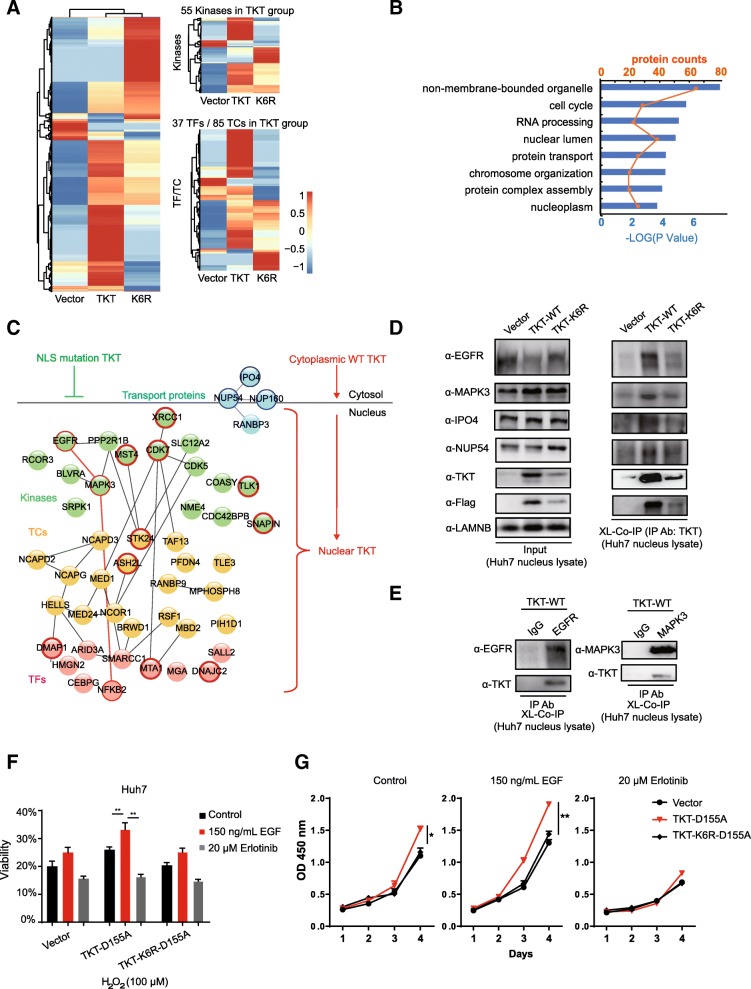
Proteomics research showed that nuclear TKT interacts with transcription factors, including EGFR, and may regulate the stress response and cell activation processes. **a** Heat maps of the identified proteins in the 3 stable cell lines. Left: total identified proteins; Upper right: identified kinases, Bottom right: identified transcription factors and co-regulators (TFs/TCs). **b** GO annotation analysis of the 243 TKT-WT unique proteins (TKT/Vector > 5 Folds and TKT/K6R >5 Folds). Blue bar: -Log of the *p* value. **c** Protein network analysis of TKT-WT unique proteins. The cell stress response-related kinases, TCs and TFs (red edges) were identified only in the TKT-WT group. Some nuclear transport proteins were also found interacted with TKT-WT (blue edges). **d** Cross-linking Co-IP and western blot assay taking TKT as the bait protein. The vector control, TKT-WT and TKT-K6R overexpressing Huh7 cells were cross-linked and nuclei were extracted and lysed by sonication. TKT antibody was used for Co-IP. Flag (for exogenous TKT detection), EGFR, MAPK3, IPO4 and NUP54 antibodies were used for western blot assay. **e** Cross-linking Co-IP and western blot assay taking TKT as the prey protein. The TKT-WT overexpressing Huh7 cells were cross-linked and nuclei were extracted and lysed by sonication. EGFR/MAPK3 and IgG antibodies were used for Co-IP. Flag (for exogenous TKT detection), TKT antibody was used for western blot assay. **f** Viability of vector control, TKT-D155A and TKT-D155A-K6R overexpressing cell lines with or without the EGF or erlotinib treatment in Huh7 cells under oxidative stress induced by H_2_O_2_ (treated for 6 h). **g** The proliferation assay of vector control, TKT-D155A and TKT-D155A-K6R overexpressing cell lines with or without the EGF or erlotinib treatment in Huh7 cells

After in gel digestion and MS identification, we detected 2058 proteins in the TKT wild-type group, among which 55 were kinases, 37 were TFs and 85 were TCs (Fig. 7a, Additional file 6: Figure S4 F). Compared with the vector control and K6R groups, the proteins were considered as TKT-WT unique if more than a 5-fold difference was detected. As a result, 243 proteins were considered to interact with nuclear TKT (Additional file 7: Table S3). These proteins were analyzed by GO annotation cluster and multiple functions were enriched including the cell cycle, chromosome organization and protein transport (Fig. 7b). Network analysis showed that the candidate TKT interacting proteins, most of which functioned as kinases, TFs or TCs, were involved in multiple signaling transduction and transcriptional regulation pathways (Fig. 7c). The data showed us key members of the cell stress response pathway, including epidermal growth factor receptor (EGFR), mitogen-activate protein kinase 3 (MAPK3), nuclear factor kappa B subunit 2 (NFKB2) and so on (Fig. 7c, red outer circles), interacted with wild-type TKT but not with the NLS mutant, suggesting potential non-metabolic mechanisms of nuclear TKT for promoting the development of HCC. We also found that only wild-type TKT interacted with NLS receptor and nuclear pore complex (NPC) proteins (Fig. 7c, blue outer circles) including importin 4 (IPO4), nucleoporin 54 (NUP54) and nucleoporin (NUP160). Some of the candidate proteins interacting with nuclear TKT were then validated by independent cross-linking Co-IP and western blot. When TKT was taken as bait protein, TKT antibody was used for Co-IP. We found EGFR, MAPK3, IPO4 and NUP54 were all detected in the wild-type TKT group compared to the endogenous control and NLS mutation groups (Fig. 7d). EGFR and MAPK3 were reported to promote the proliferation [48], survival [49, 50] or invasion [51] of multiple cancer cells. The interactions between EGFR/ MAPK3 and TKT were further validated in TKT wild-type overexpressing cells taking EGFR or MAPK3 as bait proteins. EGFR/MAPK3 antibodies and IgG control were used in cross-linking Co-IP. The interacted exogenous wild-type TKT was detected in both EGFR and MAPK3 Co-IP samples (Fig. 7e).

In order to avoid interfering by the enzyme activity, the enzyme-inactivating mutant TKT-D155A and TKT-K6R-D155A overexpression cell lines was used to investigate the potential non-metabolic roles of TKT. We examine the viability of the cells against oxidative stress induced by H_2_O_2_, with or without the treatment of EGF or erlotinib. The treatment of EGF, an agonist of EGFR pathway, significantly increased the viability of HCC cells in the TKT-D155A overexpression group but not in the D155A-K6R mutant group(Fig. 7f). When treated with EGF, the proliferation rate of TKT-D155A group was significantly increased compared to the TKT-K6R-D155A group and non-treatment TKT-D155A group (Fig. 7g). Moreover, the enhanced viability and increased proliferation rate could be abolished by the treatment of EGFR inhibitor erlotinib. These results suggested a potential non-metabolic mechanism of nuclear TKT promoting the viability and proliferation of HCC cells through EGFR pathway. The activated EGFR induced by EGF could translocate into the nucleus [52], and function as a co-transcription factor and kinase to promote proliferation, invasion and survival of cancer cells [53]. Our results suggest nuclear TKT could promote the survival of HCC cell under oxidative stress via activated EGFR.

## Discussion

It is well known that during the tumorigenesis and metastasis of HCC, the cancer cells are exposed to vastly different micro-environments and stresses. Cancer cells can reprogram their metabolism to adapt to the micro-environmental changes. The best-known metabolic abnormality in cancer cells is the Warburg effect, an increase in glycolysis as well as PPP, even in the presence of oxygen [54]. As a key enzyme in the non-oxidative phase of PPP, TKT catalyzes a series of reversible reactions [55] linking PPP to glycolysis and making the non-oxidative phase a dynamic network. This endows PPP with the ability to switch the metabolic pattern to meet the different demands of cancer cells under various stress conditions [28, 30, 56]. In the present study, we verified that TKT enzymatic activity could significantly increase the level of NADPH, which is the main reductant of cancer cells. TKT was positively correlated to metastatic potential of HCC cells and predicted a poor prognosis in HCC patients. Using in vivo and in vitro experiments, we also validated that HCC cells with high level of TKT achieved a more flexible metabolic network, resulting in enhanced survival, proliferation and metastasis.

Remarkably, our study showed that the inhibition of the enzyme activity of TKT did not entirely abolish the tumor-promoting effects in HCC cells. In addition, unlike other key enzymes in PPP, TKT showed a strong nuclear localization, where the enzymatic activity would be inhibited because of the absence of the transketolase co-factor TPP [46]. These results demonstrate the possibility that nuclear TKT might play a non-metabolic role to promote the development of HCC cells. To verify this hypothesis, we first identified the NLS of TKT using a series of truncations and mutations. We found that Y4A and K6R mutations inhibited nuclear localization of TKT, and only TKT wild type could be detected in chromatin fractions. We also found that the Y4A and K6R mutations decreased the tumor-promoting effects of TKT, even in the presence of a dominant-negative mutation of the enzyme activity. Furthermore, survival analysis using clinical data showed that a high nuclear TKT level, relative to cytoplasmic TKT, was associated with impaired liver function and predicted a poor prognosis. High nuclear TKT was an independent risk factor for both OS and DFS. These results collectively support the hypothesis that TKT could enhance the malignancy of HCC cells in a non-metabolic manner.

To further investigate the mechanism of nuclear TKT in HCC cells, we developed a cross-linking Co-IP/MS strategy to detect proteins that interact with nuclear TKT. Using this method, we uncovered numbers of weak or transient protein complexes that interacted with TKT in the nucleus, including TFs/TCs, kinases and protein transport machinery. After independent verification experiments, we validated that only wild-type TKT interacted with NLS receptor IPO4, nuclear pore complex protein NUP54 and key proteins of cell stress response pathways including EGFR and MAPK3 (ERK1).

Overexpression and aberrant activity of the EGF and EGFR have been observed in various cancer types. The interaction and regulation of them is complex and may be affected by various factors. As a receptor tyrosine kinase, EGFR would be activated and dimerized by the EGF binding [57]. It would recruit multiple protein complexes like STAT3/5, E2F1, DNA-PK, PCNA et al and activate a series of signaling cascades such as Ras, Raf, MAPK3/1, NFKB, PI3K, AKT pathways et al. EGF-induced EGFR signaling pathway triggers the activation of RAS/RAF/MEK1/ERK1/2, NF-κB, PI3K/AKT/mTOR pathways [49, 51, 58–60]. In addition, as a transcriptional co-regulator and a tyrosine kinase, nuclear EGFR directly regulates the activity or expression of oncogenes like STAT1, c-Myc, cyclin D1, proliferating cell nuclear antigen (PCNA) [53]. Furthermore, nuclear EGFR is highly associated with disease progression, worse overall survival in numerous types of cancer [53]. Therefore, it is possible that TKT participated in the co-regulation of the EGFR induced activation of signaling networks involving cell proliferation, invasion, and survival against different stresses. We validated that EGF treatment significantly increased the viability and proliferation of HCC cells in the TKT enzyme-inactivating mutant overexpression group, which could be blocked by EGFR inhibitor erlotinib. These results implied that nuclear TKT might co-regulate with EGFR to promote HCC development and survival independent of TKT enzymatic activity. However, the detailed mechanism remains to be further studied.

## Conclusions

Increased PPP and glycolysis were frequently correlated to the HCC patients with more severe prognosis [61, 62]. An increasing number of enzymes has shown non-metabolic functions that contribute to the tumor-promoting effects [14, 15, 19]. Our research showed that as a key enzyme of PPP, TKT enhanced survival, growth and malignance of HCC in vitro and in vivo. We demonstrated for the first time, that in addition to the metabolic manner, TKT can promote the viability of HCC in a non-metabolic manner via its nuclear localization and EGFR pathway.

## Additional files

Additional file 1: **Table S1.** Description for the HCC cell lines. (DOCX 13 kb)

Additional file 2: **Table S2.** qPCR primers of *TKT*, *TKTL1* and *TKTL2*. (DOCX 14 kb)

Additional file 3: **Figure S1.** The expression levels of TKTL1 and TKTL2 in HCC tissues and stable cell lines. A. *TKT*, *TKTL1* and *TKTL2* mRNA levels in TCGA database. B. The protein level of TKTL1 and TKTL2 in different HCC cell lines. (PDF 164 kb)

Additional file 4: **Figure S2.** Truncations and mutations to determine the NLS of TKT. A. TKT truncations covering 2/3 of the full length from the N or C terminus showed the presence of the NLS at both the N and C termini of TKT. B-C. Truncations and mutants determined that the NLS sites of TKT were Y4 or K6 at the N-terminus and DAIA from 610 to 614 near the C-terminus. However, any mutations in DAIA destabilized TKT. (PDF 419 kb)

Additional file 5: **Figure S3.** The cell cycle and glucose consumption assays of TKT overexpression cell lines. A. Effect of TKT and TKT mutants on cell cycle distribution of Huh7 cells. B. TKT wild type overexpression promoted the transition from G0/G1 to S phase, and the NLS mutant decrease the cell cycle regulating function of TKT. C. Although TKT enzyme-inactivating mutant D155A reduced the function of regulating cell cycle, the expression of TKT-D155A still increased the percentage of S phase, suggesting there would be a non-metabolic mechanism of TKT to regulate cell cycle. D. TKT-K6R-D155A double mutation would abolish the function of regulating cell cycle. E. The glucose consumption of TKT, NLS mutation and enzyme-inactivating mutation overexpressing cell lines. (PDF 358 kb)

Additional file 6: **Figure S4.** Workflow and quantity control of the cross-linking Co-IP/MS. A. Cross-linking Co-IP/MS workflow. B. Equally overexpressed TKT wild type and TKT NLS mutation (K6R) stable cell lines along with the empty vector control group were crosslinked by formaldehyde. C. Nucleus fractions were enriched after weak power sonication. The marker of nucleus (LAMN B) could only detected in nucleus fraction. D. Wide type TKT, but not TKT NLS mutant could be detected in nucleus fractions. E. TKT antibody was used to pull down the target protein after crosslinking. F. Overlap of proteins identified in the 3 stable cell lines by MS. (PDF 177 kb)

Additional file 7: **Table S3.** The list of 243 unique proteins interacting with nuclear TKT. (DOCX 41 kb)

## Abbreviations

Co-IP/MS: Co-Immunoprecipitation combined with mass spectrometry
DFS: Disease-free survival
EGFR: Epidermal growth factor receptor
GO: Gene ontology
GSH/GSSG: Glutathione/glutathione disulfide
HCC: Hepatocellular carcinoma
IHC: Immunohistochemistry
IPO4: Importin 4
MAPK3: Mitogen-activated protein kinase 3
MS: Mass spectrometry
NFKB2: Nuclear factor kappa B subunit 2
NLS: Nuclear localization sequence
NPC: Nuclear pore complex
NUP160: Nucleoporin 160
NUP54: Nucleoporin 54
OS: Overall survival
OT: Oxythiamine
PPP: Pentose phosphate pathway
qRT-PCR: Quantitative real-time PCR
TCs: Transcription co-regulators
TFs: Transcription factors.
TKT: Transketolase
TPP: Thiamine pyrophosphate
