# Supplementary material for: Transketolase (TKT) activity and nuclear localization promote hepatocellular carcinoma in a metabolic and a non-metabolic manner
Source: J Exp Clin Cancer Res. 2019 Apr 11;38:154. doi: 10.1186/s13046-019-1131-1 (PMC6458711; doi:10.1186/s13046-019-1131-1)
Supplement: Supplementary file 1 — Table S1. Description for the HCC cell lines. (DOCX 13 kb) [file 13046_2019_1131_MOESM1_ESM.docx]

Supplemental Table 1. Description for the HCC cell lines

| Inoculation  Position | Metastasis  Indexes | Metastasis (Y/N), *p53* mutation and HBAg-positive | | | | | | |
| --- | --- | --- | --- | --- | --- | --- | --- | --- |
|  |  | HepG2 | Hep3B | Huh7 | PLC/PRF/5 | MHCC97L | MHCC97H | HCCLM3 |
| Metastasis Ability | Metastasis | N | N | N | N |  |  |  |
|  | Orthotopic (Liver) Metastasis |  |  |  |  | Y | Y | Y |
| *p53* status | | WT | Lost | Mutated | Mutated | Mutated | Mutated | Mutated |
| HBX/HBAg | | - | + | - | + | + | + | + |
| Sex | | M | M | M | M | M | M | M |
