# Supplementary material for: Transketolase (TKT) activity and nuclear localization promote hepatocellular carcinoma in a metabolic and a non-metabolic manner
Source: J Exp Clin Cancer Res. 2019 Apr 11;38:154. doi: 10.1186/s13046-019-1131-1 (PMC6458711; doi:10.1186/s13046-019-1131-1)
Supplement: Supplementary file 2 — Table S2. qPCR primers of TKT, TKTL1 and TKTL2. (DOCX 14 kb) [file 13046_2019_1131_MOESM2_ESM.docx]

Supplemental Table 2. qPRC primers of *TKT*, *TKTL1* and *TKTL2*

| Gene | Sense (5’-3’) | Antisense (5’-3’) |
| --- | --- | --- |
| *TKT* | CGCCAATACAAAGGGTATCTG | TTTCTTTCTTCAGCAGTTCGG |
| *TKTL1* | ACAAGCAGTCAGATCCAGAGA | TAGCTGGCCCTGTCGAAGTA |
| *TKTL2* | GGGACATGCTGCTCCTATCC | CGTCAACAAACGGCAATCGG |
