# Supplementary figures and images for: Transketolase (TKT) activity and nuclear localization promote hepatocellular carcinoma in a metabolic and a non-metabolic manner
Source: J Exp Clin Cancer Res. 2019 Apr 11;38:154. doi: 10.1186/s13046-019-1131-1 (PMC6458711; doi:10.1186/s13046-019-1131-1)

# Supplementary Figure 1

A

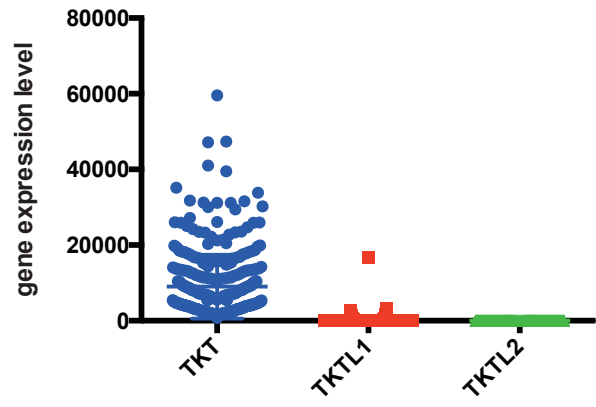

B

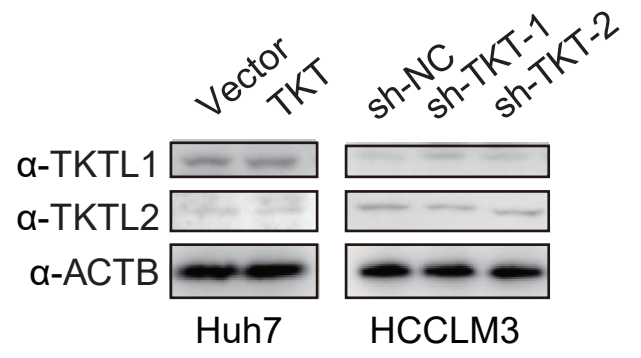

Supplement: Supplementary file 3 — Figure S1. The expression levels of TKTL1 and TKTL2 in HCC tissues and stable cell lines. A. TKT, TKTL1 and TKTL2 mRNA levels in TCGA database. B. The protein level of TKTL1 and TKTL2 in different HCC cell lines. (PDF 164 kb) [file 13046_2019_1131_MOESM3_ESM.pdf]

# Supplementary Figure 3

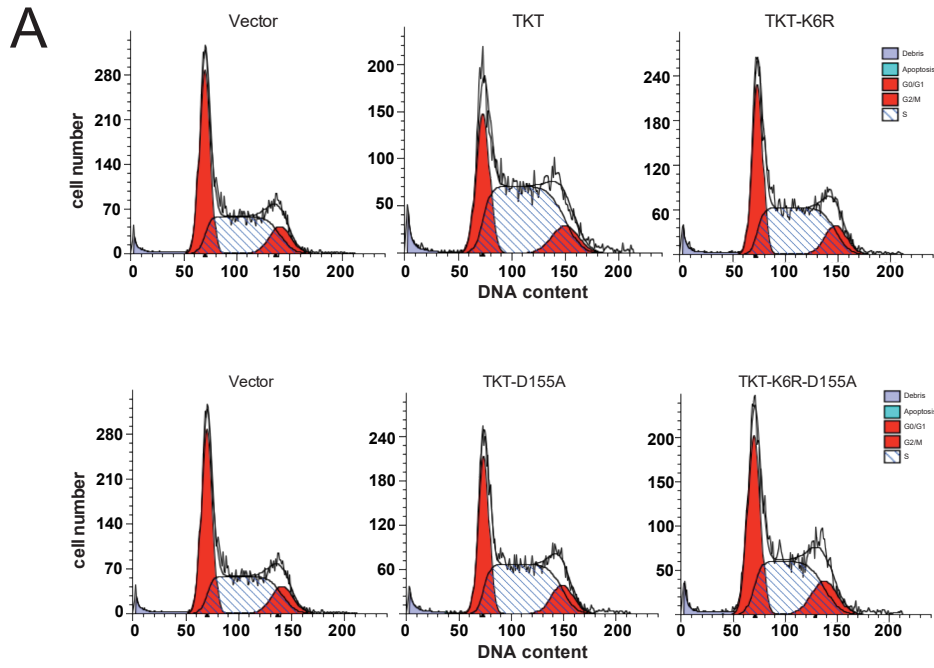

**B**

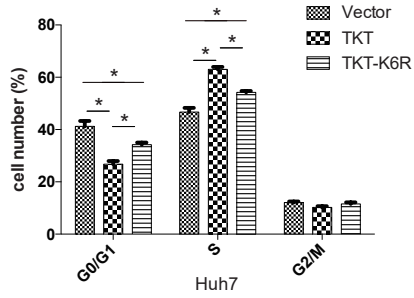

**C**

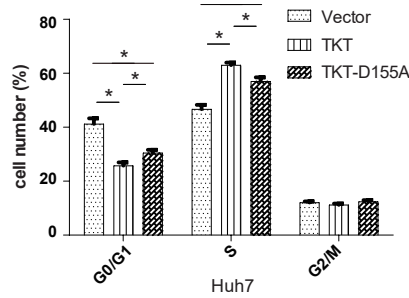

**D**

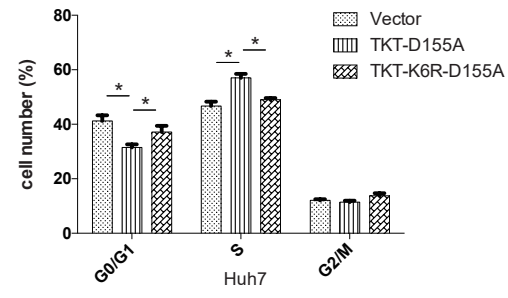

**E**

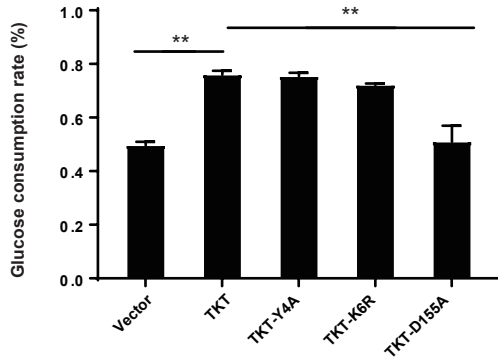

Supplement: Supplementary file 5 — Figure S3. The cell cycle and glucose consumption assays of TKT overexpression cell lines. A. Effect of TKT and TKT mutants on cell cycle distribution of Huh7 cells. B. TKT wild type overexpression promoted the transition from G0/G1 to S phase, and the NLS mutant decrease the cell cycle regulating function of TKT. C. Although TKT enzyme-inactivating mutant D155A reduced the function of regulating cell cycle, the expression of TKT-D155A still increased the percentage of S phase, suggesting there would be a non-metabolic mechanism of TKT to regulate cell cycle. D. TKT-K6R-D155A double mutation would abolish the function of regulating cell cycle. E. The glucose consumption of TKT, NLS mutation and enzyme-inactivating mutation overexpressing cell lines. (PDF 358 kb) [file 13046_2019_1131_MOESM5_ESM.pdf]

# Supplementary Figure 4

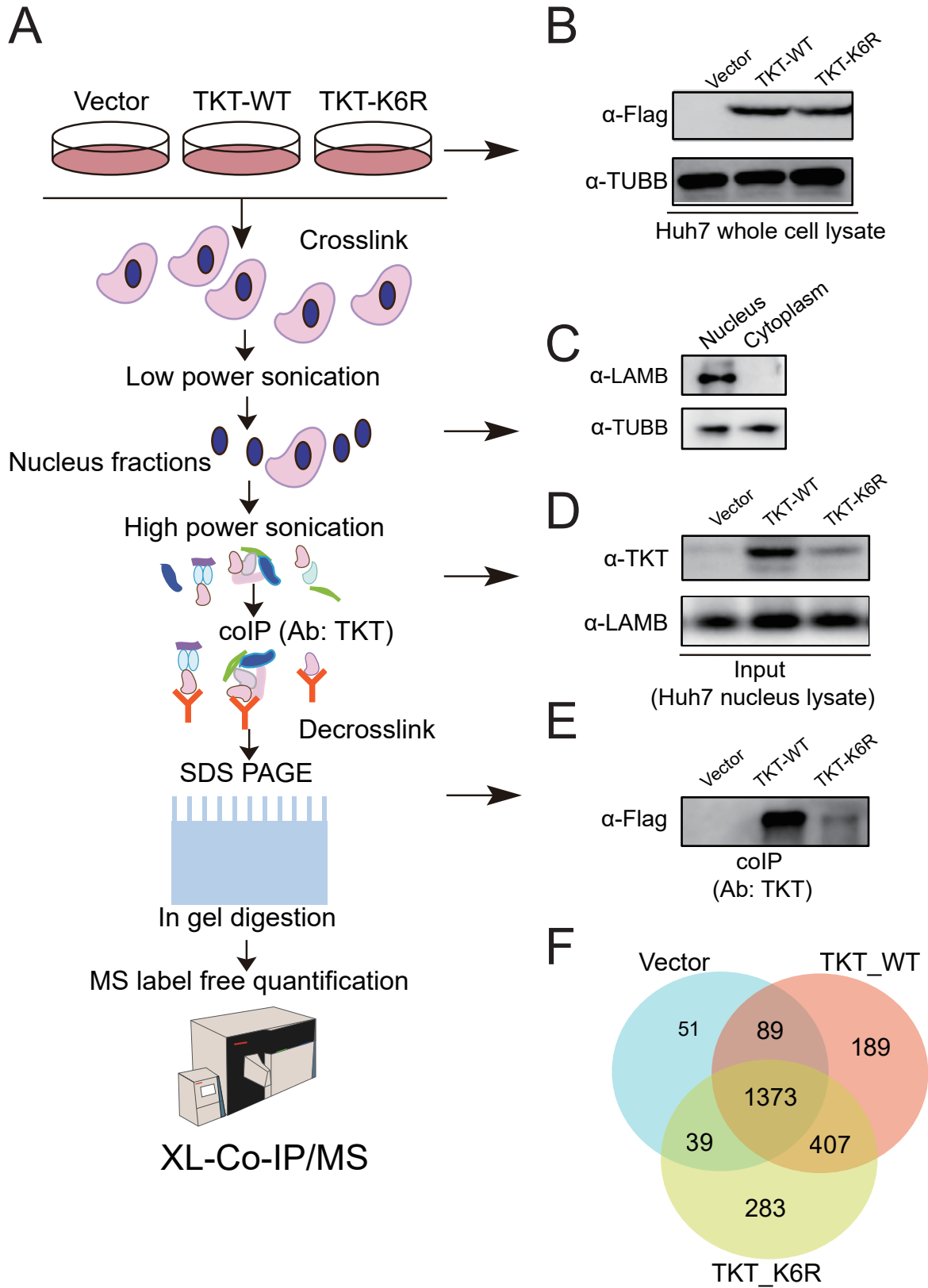

Supplement: Supplementary file 6 — Figure S4. Workflow and quantity control of the cross-linking Co-IP/MS. A. Cross-linking Co-IP/MS workflow. B. Equally overexpressed TKT wild type and TKT NLS mutation (K6R) stable cell lines along with the empty vector control group were crosslinked by formaldehyde. C. Nucleus fractions were enriched after weak power sonication. The marker of nucleus (LAMN B) could only detected in nucleus fraction. D. Wide type TKT, but not TKT NLS mutant could be detected in nucleus fractions. E. TKT antibody was used to pull down the target protein after crosslinking. F. Overlap of proteins identified in the 3 stable cell lines by MS. (PDF 177 kb) [file 13046_2019_1131_MOESM6_ESM.pdf]
