# Supplementary material for: Transketolase (TKT) activity and nuclear localization promote hepatocellular carcinoma in a metabolic and a non-metabolic manner
Source: J Exp Clin Cancer Res. 2019 Apr 11;38:154. doi: 10.1186/s13046-019-1131-1 (PMC6458711; doi:10.1186/s13046-019-1131-1)
Supplement: Supplementary file 4 — Figure S2. Truncations and mutations to determine the NLS of TKT. A. TKT truncations covering 2/3 of the full length from the N or C terminus showed the presence of the NLS at both the N and C termini of TKT. B-C. Truncations and mutants determined that the NLS sites of TKT were Y4 or K6 at the N-terminus and DAIA from 610 to 614 near the C-terminus. However, any mutations in DAIA destabilized TKT. (PDF 419 kb) [file 13046_2019_1131_MOESM4_ESM.pdf]

Supplementary Figure 2

A

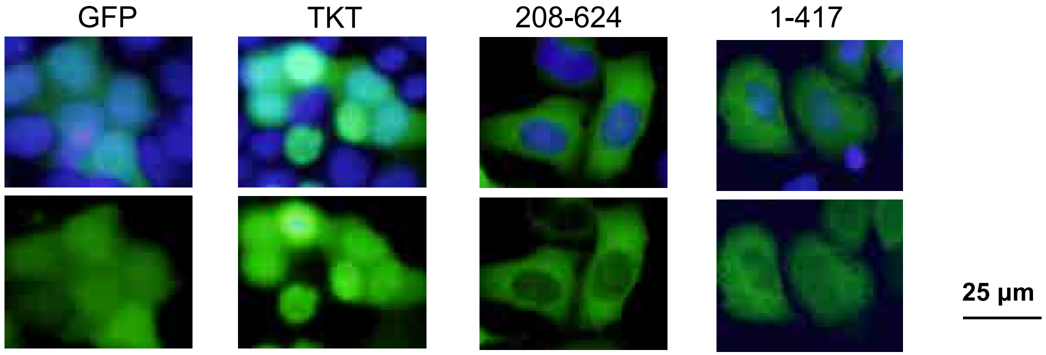

B

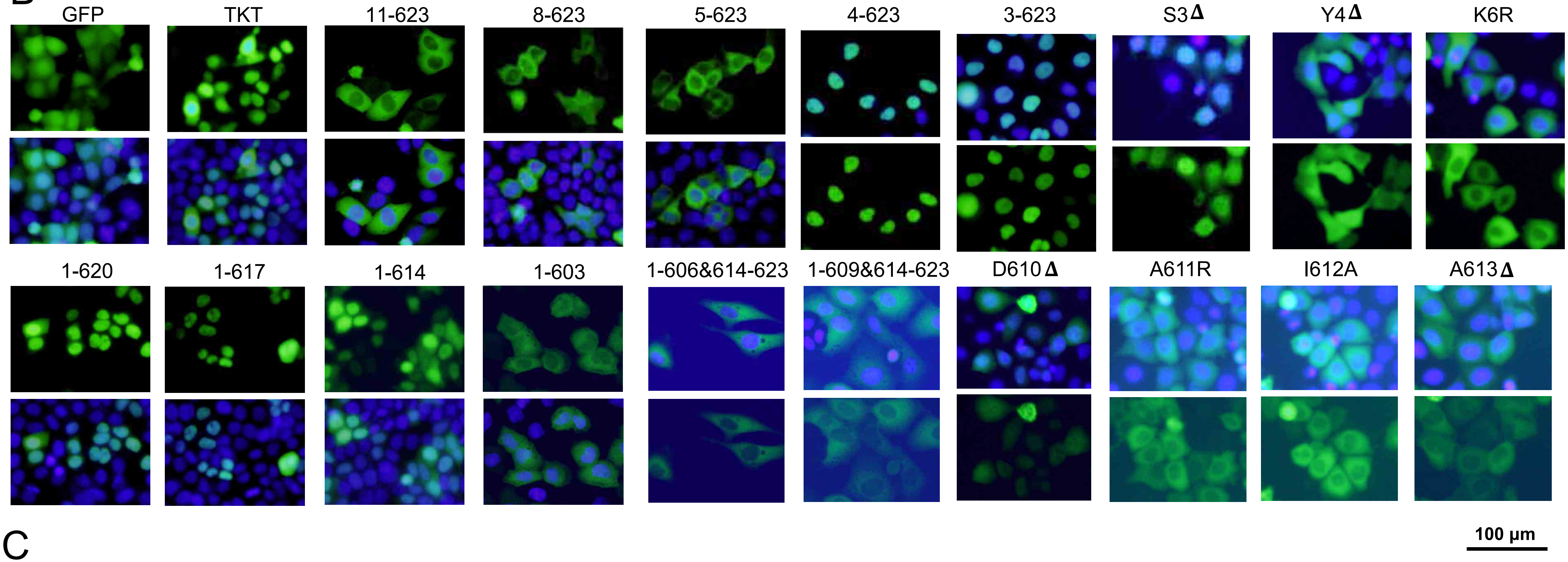

C

MESYHKPDQQKLQALKDTANRLRISSIQATTAAGSGHPTSCCSAAEIMAVLFFHTMRYKSQDPRNPHNDRFVLSKGHAAPILYAVWAEAGFLAEAELLNLRKISSDLDGHP
VPKQAFTDVATGSLGQGLGAACGMAYTGKYFDKASYRVYCLLGDGELSEGSVWEAMAFASIYKLDNLVAILDINRLGQSDPAPLQHQMDIYQKRCEAFGWHAIIVDGHSV
EELCKAFGQAKHQPTAIIAKTFKGRGITGVEDKESWHGKPLPKNMAEQIIQEIYSQIQSKKKILATPPQEDAPSVDIANIRMPSLPSYKVGDKIATRKAYGQALAKLGHASDRI
IALDGDTKNSTFSEIFKKEHPDRFIECYIAEQNMVSIAVGCATRNRTVPFCSTFAAFFTRAFDQIRMAAISESNINLCGSHCGVSIGEDGPSQMALEDLAMFRSVPTSTVFYP
SDGVATEKAVELAANTKGICFIRTSRPENAIIYNNNEDFQVGQAKVVLKSKDDQVTVIGAGVTLHEALAAAELLKKEKINIRVLDPFTIKPLDRKLILDSARATKGRILTVEDHY
YEGGIGEAVSSAVVGEPGITVTHLAVNRVPRSGKPAELLKMFGIDRDAIAQAVRGLITKA

Unstable
