# Supplementary material for: Transketolase (TKT) activity and nuclear localization promote hepatocellular carcinoma in a metabolic and a non-metabolic manner
Source: J Exp Clin Cancer Res. 2019 Apr 11;38:154. doi: 10.1186/s13046-019-1131-1 (PMC6458711; doi:10.1186/s13046-019-1131-1)
Supplement: Supplementary file 7 — Table S3. The list of 243 unique proteins interacting with nuclear TKT. (DOCX 41 kb) [file 13046_2019_1131_MOESM7_ESM.docx]

Supplementary Table 3. The list of 243 unique proteins interacting with nuclear TKT.

| ProteinIDs | geneName | geneIdNum | Vector_Intensity | TKT_Intensity | K6R_Intensity | Vector_iBAQ | TKT_iBAQ | K6R_iBAQ | TF.or.TC | kinase |
| --- | --- | --- | --- | --- | --- | --- | --- | --- | --- | --- |
| 387157878 | NFKB2 | 4791 | 6440300 | 72314000 | 0 | 157080 | 1763700 | 0 | TF | NA |
| 62460637 | IPO4 | 79711 | 584760 | 35675000 | 4784400 | 11245 | 686050 | 92007 | NA | NA |
| 29725609 | EGFR | 1956 | 3128200 | 15992000 | 1863100 | 52136 | 266530 | 31052 | NA | yes |
| 158138507 | MAPK3 | 5595 | 0 | 10808000 | 0 | 0 | 540380 | 0 | NA | yes |
| 26051237 | NUP54 | 53371 | 0 | 10004000 | 0 | 0 | 370520 | 0 | NA | NA |
| 54859722 | NUP160 | 23279 | 0 | 2123100 | 0 | 0 | 35384 | 0 | NA | NA |
| 134142826 | PCM1 | 5108 | 39043000 | 320400000 | 10249000 | 453980 | 3725600 | 119180 | NA | NA |
| 11761629 | FGA | 2243 | 4807200 | 202400000 | 37873000 | 145670 | 6133300 | 1147700 | NA | NA |
| 190014578 | SACM1L | 22908 | 0 | 195640000 | 0 | 0 | 5589700 | 0 | NA | NA |
| 5174539 | MDH1 | 4190 | 20857000 | 104870000 | 15609000 | 1390400 | 6991100 | 1040600 | NA | NA |
| 69354671 | ABCF1 | 23 | 11756000 | 94049000 | 18243000 | 317730 | 2541900 | 493050 | NA | NA |
| 119703753 | KRT6B | 3854 | 7985700 | 90951000 | 0 | 266190 | 3031700 | 0 | NA | NA |
| 39780588 | TSR1 | 55720 | 15424000 | 82748000 | 13608000 | 467400 | 2507500 | 412380 | NA | NA |
| 24430192 | KRT16 | 3868 | 0 | 76724000 | 0 | 0 | 2645600 | 0 | NA | NA |
| 21914927 | HELLS | 3070 | 12836000 | 73134000 | 0 | 320910 | 1828400 | 0 | CR | NA |
| 73623030 | CPT1A | 1374 | 10951000 | 63090000 | 7710600 | 295980 | 1705100 | 208390 | NA | NA |
| 4759264 | COPS2 | 9318 | 0 | 62983000 | 12309000 | 0 | 2999200 | 586160 | NA | NA |
| 4503423 | DUT | 1854 | 6377700 | 59736000 | 0 | 637770 | 5973600 | 0 | NA | NA |
| 224586884 | YBX3 | 8531 | 10218000 | 55258000 | 3192800 | 1021800 | 5525800 | 319280 | NA | NA |
| 4502743 | CDK7 | 1022 | 5319100 | 54475000 | 0 | 279950 | 2867100 | 0 | NA | NA |
| 188536047 | SMARCC1 | 6599 | 7426400 | 53253000 | 10429000 | 151560 | 1086800 | 212850 | TF | NA |
| 21359854 | RABGGTB | 5876 | 2960800 | 53196000 | 5239200 | 197390 | 3546400 | 349280 | NA | NA |
| 7661950 | CTR9 | 9646 | 4105400 | 52972000 | 9032100 | 70782 | 913310 | 155730 | NA | NA |
| 5031839 | KRT6A | 3853 | 10249000 | 52308000 | 5117700 | 341620 | 1743600 | 170590 | NA | NA |
| 34878777 | RNF20 | 56254 | 9911100 | 50531000 | 5746900 | 187000 | 953420 | 108430 | NA | NA |
| 4826862 | NME4 | 4833 | 5541700 | 49904000 | 0 | 461810 | 4158700 | 0 | NA | NA |
| 38570062 | GET4 | 51608 | 0 | 48089000 | 3620100 | 0 | 3434900 | 258580 | NA | NA |
| 56243533 | SDF2L1 | 23753 | 0 | 46164000 | 6813600 | 0 | 3847000 | 567800 | NA | NA |
| 4502891 | CLNS1A | 1207 | 0 | 45301000 | 0 | 0 | 5662700 | 0 | NA | NA |
| 21700763 | HN1L | 90861 | 3198100 | 41763000 | 0 | 290740 | 3796700 | 0 | NA | NA |
| 28416940 | SBDS | 51119 | 8204900 | 41218000 | 0 | 482640 | 2424600 | 0 | NA | NA |
| 21361633 | LRRC40 | 55631 | 0 | 38218000 | 3678000 | 0 | 1005700 | 96790 | NA | NA |
| 5031749 | HMGN2 | 3151 | 5642000 | 37290000 | 0 | 2821000 | 18645000 | 0 | TF | NA |
| 117647226 | TRMT10C | 54931 | 1267700 | 37015000 | 4869900 | 48756 | 1423700 | 187300 | NA | NA |
| 115298682 | PRRC2C | 23215 | 0 | 35890000 | 0 | 0 | 358900 | 0 | NA | NA |
| 7706751 | TUBG2 | 27175 | 0 | 35556000 | 0 | 0 | 1693100 | 0 | NA | NA |
| 255982614 | DHX16 | 8449 | 4383000 | 34107000 | 0 | 87660 | 682130 | 0 | NA | NA |
| 359465572 | BLVRA | 644 | 3094800 | 33886000 | 0 | 193430 | 2117900 | 0 | NA | yes |
| 17402893 | PSAT1 | 29968 | 4140500 | 33317000 | 6458600 | 207020 | 1665900 | 322930 | NA | NA |
| 318037203 | PDZK1 | 5174 | 1521300 | 33308000 | 0 | 52459 | 1148500 | 0 | NA | NA |
| 237681109 | ATP1A1 | 476 | 0 | 31995000 | 4026900 | 0 | 710990 | 89488 | NA | NA |
| 4507215 | SRP54 | 6729 | 0 | 30913000 | 5679900 | 0 | 1030400 | 189330 | NA | NA |
| 23199995 | WBSCR22 | 114049 | 3738400 | 30631000 | 3922100 | 339860 | 2784700 | 356550 | NA | NA |
| 238859597 | RBM34 | 23029 | 5095200 | 30131000 | 0 | 188710 | 1116000 | 0 | NA | NA |
| 13699256 | PPP1R8 | 5511 | 2507000 | 29379000 | 5239300 | 179070 | 2098500 | 374230 | NA | NA |
| 63253298 | SRM | 6723 | 0 | 27960000 | 0 | 0 | 1864000 | 0 | NA | NA |
| 31044432 | LEMD2 | 221496 | 0 | 27491000 | 0 | 0 | 916370 | 0 | NA | NA |
| 7661832 | SSU72 | 29101 | 0 | 27421000 | 0 | 0 | 2492800 | 0 | NA | NA |
| 223633988 | KIAA1671 | 85379 | 0 | 26576000 | 0 | 0 | 237280 | 0 | NA | NA |
| 38788319 | GNL1 | 2794 | 0 | 25711000 | 4613600 | 0 | 829390 | 148830 | NA | NA |
| 313747586 | EPB41L2 | 2037 | 3826200 | 25229000 | 0 | 81408 | 536780 | 0 | NA | NA |
| 194733742 | NELFA | 7469 | 2658000 | 24977000 | 0 | 120820 | 1135300 | 0 | NA | NA |
| 7657315 | LSM3 | 27258 | 0 | 24182000 | 0 | 0 | 8060700 | 0 | NA | NA |
| 6005862 | MRPL3 | 11222 | 0 | 23060000 | 0 | 0 | 1213700 | 0 | NA | NA |
| 7706645 | PPME1 | 51400 | 0 | 22946000 | 0 | 0 | 1349700 | 0 | NA | NA |
| 117553580 | AP3D1 | 8943 | 2625200 | 22940000 | 0 | 51475 | 449790 | 0 | NA | NA |
| 294774576 | PPP2R1B | 5519 | 0 | 22897000 | 0 | 0 | 954050 | 0 | CR | NA |
| 156766047 | GIGYF2 | 26058 | 1472600 | 22800000 | 0 | 28874 | 447050 | 0 | NA | NA |
| 4504615 | IGFBP1 | 3484 | 0 | 22718000 | 0 | 0 | 1747600 | 0 | NA | NA |
| 13699824 | KIF11 | 3832 | 0 | 22680000 | 0 | 0 | 343640 | 0 | NA | NA |
| 7657585 | SLC25A15 | 10166 | 0 | 22607000 | 3870300 | 0 | 1412900 | 241890 | NA | NA |
| 40254446 | CUL5 | 8065 | 0 | 22419000 | 3038000 | 0 | 487380 | 66044 | NA | NA |
| 4504067 | GOT1 | 2805 | 0 | 21109000 | 3401000 | 0 | 959480 | 154590 | NA | NA |
| 21359945 | NCAPG | 64151 | 0 | 20990000 | 0 | 0 | 446600 | 0 | CR | NA |
| 62241003 | CMYA5 | 202333 | 0 | 19870000 | 0 | 0 | 99349 | 0 | NA | NA |
| 189163542 | SERPINA1 | 5265 | 0 | 19711000 | 3673700 | 0 | 895940 | 166990 | NA | NA |
| 41581463 | FNBP1L | 54874 | 0 | 19572000 | 0 | 0 | 652400 | 0 | NA | NA |
| 194097489 | RANBP3 | 8498 | 0 | 18964000 | 0 | 0 | 862000 | 0 | NA | NA |
| 282165814 | TMED5 | 50999 | 0 | 18961000 | 3568400 | 0 | 1896100 | 356840 | NA | NA |
| 20149304 | ISY1 | 57461 | 3403000 | 18084000 | 0 | 243070 | 1291700 | 0 | NA | NA |
| 4506749 | RRM1 | 6240 | 0 | 17960000 | 0 | 0 | 408190 | 0 | NA | NA |
| 90819233 | MLLT4 | 4301 | 0 | 17336000 | 0 | 0 | 206380 | 0 | NA | NA |
| 4557325 | APOE | 348 | 0 | 17089000 | 0 | 0 | 776790 | 0 | NA | NA |
| 345441819 | KIF2A | 3796 | 3153800 | 16765000 | 875290 | 92760 | 493080 | 25744 | NA | NA |
| 4502395 | BECN1 | 8678 | 2804500 | 16697000 | 0 | 127480 | 758960 | 0 | NA | NA |
| 190684675 | XRCC1 | 7515 | 0 | 16543000 | 0 | 0 | 570460 | 0 | NA | yes |
| 4504169 | GSS | 2937 | 3099800 | 15722000 | 0 | 114810 | 582310 | 0 | NA | NA |
| 33469964 | SUGP1 | 57794 | 0 | 15704000 | 0 | 0 | 475880 | 0 | NA | NA |
| 4885193 | ARID3A | 1820 | 0 | 15610000 | 0 | 0 | 624410 | 0 | TF | NA |
| 7706025 | PDZD11 | 51248 | 0 | 15496000 | 0 | 0 | 2213800 | 0 | NA | NA |
| 23111032 | SNX1 | 6642 | 0 | 15175000 | 0 | 0 | 689770 | 0 | NA | NA |
| 12408677 | PFDN4 | 5203 | 0 | 15032000 | 0 | 0 | 5010700 | 0 | CR | NA |
| 14269586 | MRPS26 | 64949 | 0 | 14827000 | 0 | 0 | 1347900 | 0 | NA | NA |
| 4503421 | DUSP9 | 1852 | 0 | 14593000 | 0 | 0 | 858400 | 0 | NA | NA |
| 20127448 | POLA2 | 23649 | 0 | 14396000 | 0 | 0 | 599810 | 0 | NA | NA |
| 255918141 | CHAMP1 | 283489 | 1953000 | 14247000 | 0 | 32016 | 233560 | 0 | NA | NA |
| 5032009 | PYGM | 5837 | 0 | 14044000 | 0 | 0 | 280890 | 0 | NA | NA |
| 7706343 | FAM96B | 51647 | 0 | 13955000 | 2142600 | 0 | 2325800 | 357090 | NA | NA |
| 312176435 | SMAP2 | 64744 | 0 | 13435000 | 0 | 0 | 1221400 | 0 | NA | NA |
| 38569475 | MTX1 | 4580 | 0 | 13368000 | 0 | 0 | 534740 | 0 | NA | NA |
| 23308579 | PTGES3 | 10728 | 0 | 13319000 | 0 | 0 | 1479900 | 0 | NA | NA |
| 163792194 | TRMT1L | 81627 | 0 | 13290000 | 0 | 0 | 332250 | 0 | NA | NA |
| 16445438 | BRWD1 | 54014 | 0 | 13277000 | 0 | 0 | 117490 | 0 | CR | NA |
| 5730085 | DYNLT1 | 6993 | 0 | 13173000 | 0 | 0 | 2195500 | 0 | NA | NA |
| 24431933 | RTN4 | 57142 | 0 | 13168000 | 0 | 0 | 1097400 | 0 | NA | NA |
| 256542310 | DNAH17 | 8632 | 0 | 13070000 | 0 | 0 | 51255 | 0 | NA | NA |
| 62988355 | CCDC58 | 131076 | 0 | 12787000 | 0 | 0 | 1826800 | 0 | NA | NA |
| 256017159 | MGA | 23269 | 2238900 | 12723000 | 0 | 15026 | 85389 | 0 | TF | NA |
| 71772942 | AP1G1 | 164 | 0 | 12600000 | 0 | 0 | 359990 | 0 | NA | NA |
| 5031593 | ARPC5 | 10092 | 0 | 12562000 | 0 | 0 | 1256200 | 0 | NA | NA |
| 93277074 | RPP25 | 54913 | 1814600 | 12278000 | 0 | 201630 | 1364300 | 0 | NA | NA |
| 110611228 | UTRN | 7402 | 0 | 11658000 | 0 | 0 | 66997 | 0 | NA | NA |
| 256818748 | RCOR3 | 55758 | 0 | 11626000 | 0 | 0 | 553620 | 0 | CR | NA |
| 134304853 | ARHGAP29 | 9411 | 0 | 11440000 | 0 | 0 | 154600 | 0 | NA | NA |
| 178056552 | NCAPD2 | 9918 | 0 | 10998000 | 0 | 0 | 146640 | 0 | CR | NA |
| 16933567 | RAB8A | 4218 | 0 | 10930000 | 0 | 0 | 840760 | 0 | NA | NA |
| 115527080 | MTA1 | 9112 | 0 | 10752000 | 0 | 0 | 244350 | 0 | TF | NA |
| 39812378 | RANBP9 | 10048 | 0 | 10681000 | 0 | 0 | 464380 | 0 | CR | NA |
| 7706557 | C9orf78 | 51759 | 0 | 10664000 | 0 | 0 | 710930 | 0 | NA | NA |
| 22538461 | NCOR1 | 9611 | 0 | 10436000 | 0 | 0 | 80281 | 0 | CR | NA |
| 13775200 | SF3B5 | 83443 | 0 | 10145000 | 0 | 0 | 2028900 | 0 | NA | NA |
| 109255228 | CEP170 | 9859 | 0 | 10045000 | 0 | 0 | 119580 | 0 | NA | NA |
| 23943880 | MRI1 | 84245 | 0 | 10019000 | 0 | 0 | 527300 | 0 | NA | NA |
| 38044112 | CLIP1 | 6249 | 0 | 9875400 | 0 | 0 | 126610 | 0 | NA | NA |
| 109633028 | MST4 | 51765 | 0 | 9530300 | 0 | 0 | 476510 | 0 | NA | NA |
| 4503165 | CUL3 | 8452 | 0 | 9329500 | 0 | 0 | 212030 | 0 | NA | NA |
| 7661532 | NOB1 | 28987 | 0 | 9175000 | 0 | 0 | 398910 | 0 | NA | NA |
| 31083243 | PPP2R5C | 5527 | 0 | 8788400 | 0 | 0 | 418490 | 0 | NA | NA |
| 34101288 | CPSF2 | 53981 | 0 | 8784600 | 0 | 0 | 204290 | 0 | NA | NA |
| 41055989 | MPHOSPH8 | 54737 | 0 | 8706600 | 0 | 0 | 272080 | 0 | CR | NA |
| 259013556 | FXR2 | 9513 | 0 | 8696300 | 0 | 0 | 248470 | 0 | NA | NA |
| 134152683 | TMEM214 | 54867 | 0 | 8625000 | 0 | 0 | 253680 | 0 | NA | NA |
| 304555614 | BRAT1 | 221927 | 0 | 8602700 | 0 | 0 | 191170 | 0 | NA | NA |
| 211971038 | TLK1 | 9874 | 0 | 8522700 | 0 | 0 | 266330 | 0 | NA | NA |
| 24308295 | GRPEL1 | 80273 | 0 | 8455800 | 0 | 0 | 563720 | 0 | NA | NA |
| 219842325 | MAPRE2 | 10982 | 0 | 8310200 | 0 | 0 | 554010 | 0 | NA | NA |
| 32171175 | BUD31 | 8896 | 0 | 8118400 | 0 | 0 | 1014800 | 0 | NA | NA |
| 124256489 | CRNKL1 | 51340 | 0 | 8086500 | 0 | 0 | 161730 | 0 | NA | NA |
| 6631085 | DNAJB4 | 11080 | 0 | 8008900 | 0 | 0 | 444940 | 0 | NA | NA |
| 166795250 | KIF2C | 11004 | 0 | 7700600 | 0 | 0 | 208120 | 0 | NA | NA |
| 189027129 | PDE12 | 201626 | 0 | 7668300 | 0 | 0 | 306730 | 0 | NA | NA |
| 75709204 | SAR1B | 51128 | 0 | 7377800 | 0 | 0 | 737780 | 0 | NA | NA |
| 256542306 | CDK5 | 1020 | 0 | 7280400 | 0 | 0 | 455030 | 0 | NA | NA |
| 50345877 | ATE1 | 11101 | 0 | 7169100 | 0 | 0 | 341390 | 0 | NA | NA |
| 217272839 | CHDH | 55349 | 0 | 6920900 | 0 | 0 | 203560 | 0 | NA | NA |
| 78000213 | DMAP1 | 55929 | 0 | 6907600 | 0 | 0 | 276310 | 0 | TF | NA |
| 214010191 | RNASEH2B | 79621 | 0 | 6513200 | 0 | 0 | 501020 | 0 | NA | NA |
| 19526773 | ATG3 | 64422 | 0 | 6450700 | 0 | 0 | 586420 | 0 | NA | NA |
| 22325364 | UBAP2 | 55833 | 0 | 6320700 | 0 | 0 | 217950 | 0 | NA | NA |
| 154448890 | THOC1 | 9984 | 0 | 6312100 | 0 | 0 | 175340 | 0 | NA | NA |
| 13129110 | WDR77 | 79084 | 0 | 6249800 | 0 | 0 | 568170 | 0 | NA | NA |
| 7661734 | DCPS | 28960 | 0 | 6189700 | 0 | 0 | 343870 | 0 | NA | NA |
| 4507751 | TYMS | 7298 | 0 | 6013400 | 0 | 0 | 375840 | 0 | NA | NA |
| 40288284 | NUDT1 | 4521 | 0 | 5937800 | 0 | 0 | 848260 | 0 | NA | NA |
| 193788632 | DNAJC2 | 27000 | 0 | 5928600 | 0 | 0 | 211740 | 0 | TF | NA |
| 27734984 | HYPK | 25764 | 0 | 5780100 | 0 | 0 | 963360 | 0 | NA | NA |
| 4503323 | DHFR | 1719 | 0 | 5685900 | 0 | 0 | 437370 | 0 | NA | NA |
| 110227603 | COASY | 80347 | 0 | 5654500 | 0 | 0 | 257020 | 0 | NA | NA |
| 4505117 | MBD2 | 8932 | 0 | 5623700 | 0 | 0 | 330800 | 0 | CR | NA |
| 387912535 | ASH2L | 9070 | 0 | 5619700 | 0 | 0 | 208140 | 0 | CR | NA |
| 32528286 | ACOT7 | 11332 | 0 | 5597800 | 0 | 0 | 310990 | 0 | NA | NA |
| 31542652 | CCDC94 | 55702 | 0 | 5544500 | 0 | 0 | 346530 | 0 | NA | NA |
| 354681993 | PAPOLA | 10914 | 0 | 5473400 | 0 | 0 | 421030 | 0 | NA | NA |
| 115527097 | CDC42BPB | 9578 | 0 | 5442200 | 0 | 0 | 65569 | 0 | NA | NA |
| 8923598 | PIH1D1 | 55011 | 0 | 5427000 | 0 | 0 | 319240 | 0 | CR | NA |
| 300244514 | YAF2 | 10138 | 0 | 5257800 | 0 | 0 | 1314400 | 0 | NA | NA |
| 7705696 | TXNDC12 | 51060 | 0 | 5256600 | 0 | 0 | 525660 | 0 | NA | NA |
| 7661636 | SYF2 | 25949 | 0 | 5249600 | 0 | 0 | 583290 | 0 | NA | NA |
| 49574529 | UTP6 | 55813 | 0 | 5228800 | 0 | 0 | 130720 | 0 | NA | NA |
| 10863977 | LSM2 | 57819 | 0 | 5138000 | 0 | 0 | 856330 | 0 | NA | NA |
| 374253823 | SLC12A2 | 6558 | 0 | 4896200 | 0 | 0 | 102000 | 0 | NA | yes |
| 145701028 | NDUFAF7 | 55471 | 0 | 4833600 | 0 | 0 | 230170 | 0 | NA | NA |
| 142976729 | HSD17B11 | 51170 | 0 | 4829900 | 0 | 0 | 284110 | 0 | NA | NA |
| 5453990 | PSME1 | 5720 | 0 | 4796100 | 0 | 0 | 319740 | 0 | NA | NA |
| 40217812 | TBRG4 | 9238 | 0 | 4759500 | 0 | 0 | 148740 | 0 | NA | NA |
| 7706333 | MRPL48 | 51642 | 0 | 4711200 | 0 | 0 | 471120 | 0 | NA | NA |
| 21687129 | NUDCD2 | 134492 | 0 | 4680800 | 0 | 0 | 668680 | 0 | NA | NA |
| 94557305 | MRPL19 | 9801 | 0 | 4673000 | 0 | 0 | 259610 | 0 | NA | NA |
| 4505067 | MAD2L1 | 4085 | 0 | 4639700 | 0 | 0 | 331410 | 0 | NA | NA |
| 56713256 | CCBL2 | 56267 | 0 | 4584500 | 0 | 0 | 218310 | 0 | NA | NA |
| 324021716 | PPP5C | 5536 | 0 | 4554900 | 0 | 0 | 168700 | 0 | NA | NA |
| 209447096 | TMEM2 | 23670 | 0 | 4478100 | 0 | 0 | 58157 | 0 | NA | NA |
| 7305303 | NCKAP1 | 10787 | 0 | 4469200 | 0 | 0 | 73265 | 0 | NA | NA |
| 8923415 | 5-Mar | 54708 | 0 | 4463700 | 0 | 0 | 318840 | 0 | NA | NA |
| 503777067 | POLE3 | 54107 | 0 | 4393600 | 0 | 0 | 549200 | 0 | NA | NA |
| 8922301 | WDR70 | 55100 | 0 | 4380000 | 0 | 0 | 141290 | 0 | NA | NA |
| 7706367 | GINS2 | 51659 | 0 | 4332400 | 0 | 0 | 433240 | 0 | NA | NA |
| 16507200 | USP28 | 57646 | 0 | 4328800 | 0 | 0 | 75945 | 0 | NA | NA |
| 157384984 | TLE3 | 7090 | 0 | 4186600 | 0 | 0 | 116290 | 0 | CR | NA |
| 18105056 | VPS33A | 65082 | 0 | 4033800 | 0 | 0 | 115250 | 0 | NA | NA |
| 125490370 | KRT40 | 125115 | 0 | 3971800 | 0 | 0 | 233630 | 0 | NA | NA |
| 4504747 | ITGA3 | 3675 | 0 | 3963300 | 0 | 0 | 99082 | 0 | NA | NA |
| 109689720 | ACBD5 | 91452 | 0 | 3960700 | 0 | 0 | 165030 | 0 | NA | NA |
| 118343647 | HSPB11 | 51668 | 0 | 3915500 | 0 | 0 | 783090 | 0 | NA | NA |
| 22218339 | FAM110B | 90362 | 0 | 3906000 | 0 | 0 | 169820 | 0 | NA | NA |
| 336176112 | NXT2 | 55916 | 0 | 3893200 | 0 | 0 | 1297700 | 0 | NA | NA |
| 47419936 | SRPK1 | 6732 | 0 | 3836900 | 0 | 0 | 137030 | 0 | NA | yes |
| 38788333 | RSF1 | 51773 | 0 | 3756500 | 0 | 0 | 56917 | 0 | CR | NA |
| 4826649 | MRPL49 | 740 | 0 | 3715900 | 0 | 0 | 337810 | 0 | NA | NA |
| 216548445 | TRMT1 | 55621 | 0 | 3715400 | 0 | 0 | 116110 | 0 | NA | NA |
| 65288071 | TNS3 | 64759 | 0 | 3694400 | 0 | 0 | 67170 | 0 | NA | NA |
| 324711009 | XIAP | 331 | 0 | 3539500 | 0 | 0 | 107260 | 0 | NA | NA |
| 213385323 | ANAPC5 | 51433 | 0 | 3332500 | 0 | 0 | 95215 | 0 | NA | NA |
| 21071014 | EPS8L3 | 79574 | 0 | 3314400 | 0 | 0 | 110480 | 0 | NA | NA |
| 5453601 | CRTAP | 10491 | 0 | 3254000 | 0 | 0 | 130160 | 0 | NA | NA |
| 116812608 | TTC38 | 55020 | 0 | 3252500 | 0 | 0 | 135520 | 0 | NA | NA |
| 6912674 | SNAPIN | 23557 | 0 | 3250800 | 0 | 0 | 361200 | 0 | NA | yes |
| 71565154 | ADH5 | 128 | 0 | 3211800 | 0 | 0 | 160590 | 0 | NA | NA |
| 356582519 | CEBPG | 1054 | 0 | 3210900 | 0 | 0 | 3210900 | 0 | TF | NA |
| 170932494 | STX7 | 8417 | 0 | 3166400 | 0 | 0 | 287850 | 0 | NA | NA |
| 50962882 | GPATCH8 | 23131 | 0 | 3160600 | 0 | 0 | 52676 | 0 | NA | NA |
| 9966867 | EIF5A2 | 56648 | 0 | 3059500 | 0 | 0 | 382440 | 0 | NA | NA |
| 341865566 | NUSAP1 | 51203 | 0 | 3020400 | 0 | 0 | 137290 | 0 | NA | NA |
| 215490056 | MGEA5 | 10724 | 0 | 3003400 | 0 | 0 | 69846 | 0 | NA | NA |
| 95113664 | LRRC1 | 55227 | 0 | 2961900 | 0 | 0 | 113920 | 0 | NA | NA |
| 56788368 | PICALM | 8301 | 0 | 2954900 | 0 | 0 | 123120 | 0 | NA | NA |
| 94538362 | FLOT2 | 2319 | 0 | 2939600 | 0 | 0 | 113060 | 0 | NA | NA |
| 13129100 | DCTPP1 | 79077 | 0 | 2884600 | 0 | 0 | 262240 | 0 | NA | NA |
| 54607074 | YBEY | 54059 | 0 | 2854200 | 0 | 0 | 570850 | 0 | NA | NA |
| 150010639 | USP22 | 23326 | 0 | 2836300 | 0 | 0 | 118180 | 0 | NA | NA |
| 193083172 | NAF1 | 92345 | 0 | 2776000 | 0 | 0 | 213540 | 0 | NA | NA |
| 153285408 | MTTP | 4547 | 0 | 2764000 | 0 | 0 | 57584 | 0 | NA | NA |
| 74136552 | MESDC2 | 23184 | 0 | 2744200 | 0 | 0 | 274420 | 0 | NA | NA |
| 449083351 | SEC16A | 9919 | 0 | 2736600 | 0 | 0 | 32579 | 0 | NA | NA |
| 296278206 | PARD3 | 56288 | 0 | 2451700 | 0 | 0 | 44577 | 0 | NA | NA |
| 29029591 | FTSJ1 | 24140 | 0 | 2433600 | 0 | 0 | 202800 | 0 | NA | NA |
| 109150435 | ANKZF1 | 55139 | 0 | 2330100 | 0 | 0 | 68533 | 0 | NA | NA |
| 221136866 | WRAP53 | 55135 | 0 | 2314500 | 0 | 0 | 121820 | 0 | NA | NA |
| 8923458 | COMMD8 | 54951 | 0 | 2303700 | 0 | 0 | 230370 | 0 | NA | NA |
| 28559039 | MED1 | 5469 | 0 | 2282200 | 0 | 0 | 29259 | 0 | CR | NA |
| 13386490 | LIMD2 | 80774 | 0 | 2271700 | 0 | 0 | 252410 | 0 | NA | NA |
| 40254982 | FAM107B | 83641 | 0 | 2157000 | 0 | 0 | 134810 | 0 | NA | NA |
| 41349456 | PREP | 5550 | 0 | 2144900 | 0 | 0 | 45636 | 0 | NA | NA |
| 199559805 | PPP1R18 | 170954 | 0 | 2135000 | 0 | 0 | 73621 | 0 | NA | NA |
| 384871683 | MLH1 | 4292 | 0 | 2074300 | 0 | 0 | 62857 | 0 | NA | NA |
| 6912540 | NUBP2 | 10101 | 0 | 2008900 | 0 | 0 | 143490 | 0 | NA | NA |
| 150417986 | ARFGEF2 | 10564 | 0 | 1889900 | 0 | 0 | 19090 | 0 | NA | NA |
| 392583926 | MED24 | 9862 | 0 | 1808400 | 0 | 0 | 35459 | 0 | CR | NA |
| 49087132 | SALL2 | 6297 | 0 | 1756500 | 0 | 0 | 67559 | 0 | TF | NA |
| 5032155 | TAF13 | 6884 | 0 | 1743700 | 0 | 0 | 435920 | 0 | CR | NA |
| 203098816 | PDHX | 8050 | 0 | 1696800 | 0 | 0 | 70701 | 0 | NA | NA |
| 45356151 | NCAPD3 | 23310 | 0 | 1678000 | 0 | 0 | 24676 | 0 | CR | NA |
| 444299651 | CASC5 | 57082 | 0 | 1634800 | 0 | 0 | 13400 | 0 | NA | NA |
| 305855090 | RAB9A | 9367 | 0 | 1523600 | 0 | 0 | 138510 | 0 | NA | NA |
| 215490049 | MORF4L2 | 9643 | 0 | 1519200 | 0 | 0 | 89367 | 0 | NA | NA |
| 4507229 | ALDH5A1 | 7915 | 0 | 1445900 | 0 | 0 | 55610 | 0 | NA | NA |
| 39725938 | POLR3A | 11128 | 0 | 1286900 | 0 | 0 | 15140 | 0 | NA | NA |
| 30795227 | DTD1 | 92675 | 0 | 1172400 | 0 | 0 | 146550 | 0 | NA | NA |
| 293597521 | RRAS2 | 22800 | 0 | 1087500 | 0 | 0 | 120840 | 0 | NA | NA |
| 19557691 | SURF4 | 6836 | 0 | 852590 | 0 | 0 | 77508 | 0 | NA | NA |
| 24497618 | ATAD2 | 29028 | 0 | 851050 | 0 | 0 | 12334 | 0 | NA | NA |
| 110349738 | STK24 | 8428 | 0 | 709110 | 0 | 0 | 35456 | 0 | CR | yes |
| 21361716 | GOLPH3L | 55204 | 0 | 470400 | 0 | 0 | 27670 | 0 | NA | NA |
